# Cooperative Effects Drive Water Oxidation Catalysis in Cobalt Electrocatalysts through the Destabilization of Intermediates

**DOI:** 10.1021/jacs.3c11651

**Published:** 2024-03-22

**Authors:** Benjamin Moss, Katrine Louise Svane, David Nieto-Castro, Reshma R. Rao, Soren B. Scott, Cindy Tseng, Michael Sachs, Anuj Pennathur, Caiwu Liang, Louise I. Oldham, Eva Mazzolini, Lole Jurado, Gopinathan Sankar, Stephen Parry, Veronica Celorrio, Jahan M. Dawlaty, Jan Rossmeisl, J. R. Galán-Mascarós, Ifan E. L. Stephens, James R. Durrant

**Affiliations:** † Imperial College London, Molecular Sciences Research Hub (MSRH), 82 Wood Lane, London W120BZ, United Kingdom; ‡ University of Copenhagen, Universitetsparken 5, 2100 København Ø, Denmark; § Institut Català d’Investigació Química (ICIQ), Avda. Països Catalans 16, 43007, Tarragona, Spain; ∥ Department of Chemistry, University of Southern California, Los Angeles, California 90089-1062, United States; ⊥ SLAC National Accelerator Laboratory, 2575 Sand Hill Road, Menlo Park, California 94025, United States; # Diamond Light Source, Harwell Science and Innovation Campus, Fermi Ave., Didcot OX11 0D, United Kingdom; □ ICREA, Passeig Lluís Companys 23, 08010, Barcelona, Spain

## Abstract

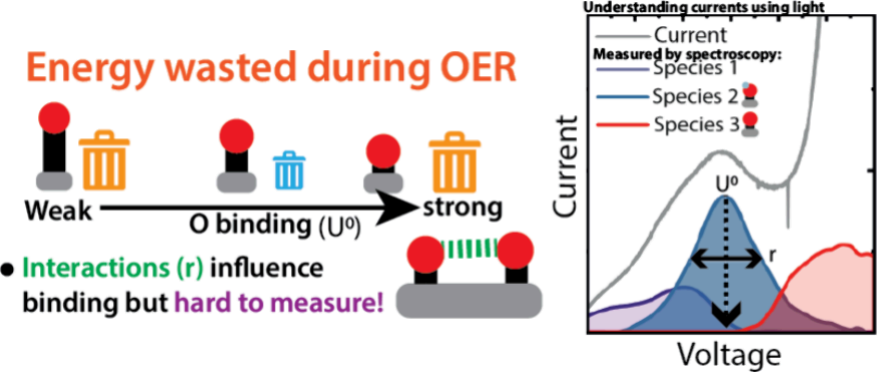

A barrier to understanding the factors driving catalysis in the oxygen evolution reaction (OER) is understanding multiple overlapping redox transitions in the OER catalysts. The complexity of these transitions obscure the relationship between the coverage of adsorbates and OER kinetics, leading to an experimental challenge in measuring activity descriptors, such as binding energies, as well as adsorbate interactions, which may destabilize intermediates and modulate their binding energies. Herein, we utilize a newly designed optical spectroelectrochemistry system to measure these phenomena in order to contrast the behavior of two electrocatalysts, cobalt oxyhydroxide (CoOOH) and cobalt–iron hexacyanoferrate (cobalt–iron Prussian blue, CoFe-PB). Three distinct optical spectra are observed in each catalyst, corresponding to three separate redox transitions, the last of which we show to be active for the OER using time-resolved spectroscopy and electrochemical mass spectroscopy. By combining predictions from density functional theory with parameters obtained from electroadsorption isotherms, we demonstrate that a destabilization of catalytic intermediates occurs with increasing coverage. In CoOOH, a strong (∼0.34 eV/monolayer) destabilization of a strongly bound catalytic intermediate is observed, leading to a potential offset between the accumulation of the intermediate and measurable O_2_ evolution. We contrast these data to CoFe-PB, where catalytic intermediate generation and O_2_ evolution onset coincide due to weaker binding and destabilization (∼0.19 eV/monolayer). By considering a correlation between activation energy and binding strength, we suggest that such adsorbate driven destabilization may account for a significant fraction of the observed OER catalytic activity in both materials. Finally, we disentangle the effects of adsorbate interactions on state coverages and kinetics to show how adsorbate interactions determine the observed Tafel slopes. Crucially, the case of CoFe-PB shows that, even where interactions are weaker, adsorption remains non-Nernstian, which strongly influences the observed Tafel slope.

## Introduction

Extracting electrons from water through the oxygen evolution reaction (OER) is a crucial element of carbon neutral fuel generation and other energy storage technologies.^1−3^ Large overpotential losses in the OER arise from kinetic barrier associated multiple proton and electron transfer steps.^3^ Lowering this barrier is key to improving the performance. However, a complete understanding of the factors needed to search for new catalysts and optimize existing ones has not yet been reached. Currently, the most powerful descriptor to search for new OER catalysts are “volcano” relations in the binding energy of surface bound catalytic intermediates. Here, the barrier for a multistep reaction, such as OER, is decided by the binding energy of the rate limiting intermediate in the catalytic cycle arising from successive oxidation steps, with the smallest barrier avoiding extremes of binding.^4^ Volcano relations are underpinned by the assumption that the free energy change of the rate limiting elementary step (i.e., binding energy) is proportional to the activation energy of the reaction (hereafter referred to as Bronsted, Evans, Polanyi (BEP) scaling).^4−8^ To optimize existing catalysts, analyses of Tafel slopes are often performed. Insight in such analyses is drawn from interpretation approaches ranging in sophistication: from detailed microkinetic models, explicitly modeling each step of the reaction, with the interconversion of intermediates following Nernst equation behavior (also known as Langmuir electroadsorption),^9^ to simpler approaches where a change in slope is assumed to be related to a change in the rate-determining step (RDS).^10^ However, all assume rate to be directly driven by potential and that an adsorbate is characterized by a single binding energy: its standard redox potential.

An increasing body of evidence suggests that binding energies of rate limiting intermediates may be modulated by the coverage of adsorbates and that such effects influence the activation energy. Changing the coverage of surface intermediates has been reported to modulate adsorbate interactions and binding energies on simple metallic surfaces.^11−14^ However, the effect of coverage driven modulations on the binding energies of oxide OER catalysts is unclear. Recently, Nong et al. provided evidence that OER on *IrO*_*x*_ in acidic media is driven primarily by adsorbate interactions.^6^ Interactions are also suggested to play a dominant role in dictating the Tafel slope, both through the BEP scaling of activation energy with binding energy and the non-Nernstian dependence of coverage on potential (known as Frumkin electroadsorption) that arises as a result of such interactions.^6^ Adsorbate interactions (suggestive of interaction influenced Tafel slopes) have also been observed by Risch et al. in amorphous, oxidic cobalt phosphate/cobalt oxyhydroxide/“CoP_i_” catalysts under neutral conditions^15^ and by our group in doped nickel oxyhydroxides under alkaline conditions.^16^ However, direct operando experimental studies of the strengths of such interactions, and how these determine observed Tafel slopes, have been limited in the literature to date.

**Figure 1:**
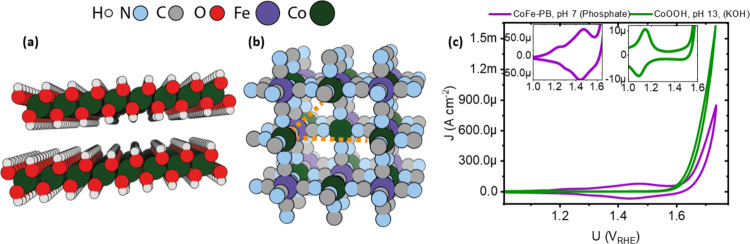
(a) The structure of Co(OH)_2_. (b) Structure of CoFe-PB. Orange dashes indicate the 7.3 Å separation of Co ions, the simplest possible defect, an Fe(CN)_6_, vacancy, which may limit interactions. We note that extensive defect ordering may lead to complex structures and may allow amorphization to occur. (c) Cyclic voltammograms (IUPAC current convention) of CoFe-PB and CoOOH measured at 1 mV s^–1^.

Without accessible and fully quantitative experimental measurements of adsorption isotherms, the interactions that they embody cannot be evaluated routinely. It is currently possible to measure adsorbate interactions on highly ordered single crystal facets^17^ and to obtain coarse indications of non-Nernstian behavior using highly specialized electrochemical cells/substrates/samples optimized for operando X-ray absorption spectroscopy.^6,15^ We have previously reported the use of spectroelectrochemical (SEC) analysis in determining the densities of reaction intermediates as a function of potential relating these to reaction kinetics in IrO_*x*_, Ni(M)OOH, and RuO_*x*_.^16,18−21^ However, the manual nature of measurements, lower sensitivity, and coarse voltage sampling of the setup used in these previous studies prevented us from accurately measuring the interactions involved.^16,19^ Herein, we overcome these problems using a custom SEC setup employing a multichannel detector, using optimized optics, and by automating the measurement to produce the high-resolution spectra with the dense voltage sampling needed to reconstruct adsorption isotherms. We measure for the first-time adsorption isotherms in complex materials; extracting both the binding energy and the strength of adsorbate interactions of the rate limiting intermediate. As a case study, we use the example of cobalt oxyhydroxide (CoOOH, Figure 1a), whose behavior we find to be dominated by strong adsorbate interactions. We contrast these results to those seen in cobalt–iron hexacyanoferrate (also known as cobalt–iron Prussian blue, CoFe-PB, Figure 1b), which exhibits weaker interactions. Despite this, we observe that the absorption isotherm of CoFe-PB cannot be explained without non-Nernstian correction. Thus, even where an electrocatalyst behaves in a more “classical” manner, the behavior of the Tafel slope cannot be understood without accounting for interactions.

In a recent review, a compelling argument has been made by Frei that the observation of an intermediate *“should not be considered sufficient to establish a mechanism without kinetic evidence of a TOF large enough to support the measured current”*.^22^ This point is pertinent in catalysts for which numerous intermediates have been reported, as without a direct measure of kinetics, a given observed intermediate may not, in fact, turn over at a sufficient rate to support the measured current/rate of O_2_ generation. The point is especially salient for CoOOH, where OER has been suggested in different studies to arise from terminal Co^4+^ oxo species and Co^3+^ superoxo species.^15,23,24^ There is further controversy regarding the nature of the rate-determining step, with separate studies suggesting the attack of Co^4+^ oxo species or the desorption of O_2_ from a Co^3+^ superoxo cluster.^23−26^ Interestingly, recent operando electron microscopy has contested these oxidation state assignments and suggested oxidation to be coupled to ingress of hydroxide.^27^ Multiple intermediates might possibly be expected in CoFe-PB, as it forms Fe(CN)_6_ vacancies (orange dashes, Figure 1b). In the simplest case, this would create unsaturated cobalt sites capable of binding water but with sufficient separation to limit interactions between sites. However, Prussian blue analogues typically show extensive defect ordering, leading to highly porous structures.^28^ Such ordering may lead to highly undercoordinated redox active^29^ metal surfaces that have been shown to be active for water oxidation in neutral and also in acidic media.^30^ CoFe-PB and other Prussian blue analogues may also yield amorphized oxyhydroxide-like surfaces, particularly when operated at high pH (pH > 13).^31,32^ To minimize such effects, we use a neutral pH electrolyte for the comparative experiments in this work and rapidly acquire spectral data on the time scale of minutes to minimize any changes to the CoFe-PB surface. We note that exact nature of the active Prussian blue surface is beyond the scope of this study, as we aim only to contrast the influence of adsorbate interactions in the two catalysts as a case study on the relation between catalyst oxidation and OER catalysis and to highlight the influence of interactions on the Tafel slope.

To overcome the possibility of measuring intermediates that do not turn over sufficiently quickly to support the observed rate of the OER, we combine our SEC measurements with time-resolved kinetic measurements of the putative intermediates in conjunction with operando electrochemical mass spectrometry measurements of the O_2_ evolution. By matching the apparent turnover frequency from SEC (calculated by simply dividing the OER current by the charge stored in the form of the rate limiting intermediate) to time-resolved kinetics, we are able to demonstrate that the intermediate we measure turns over sufficiently quickly to support current and, thus, given that this intermediate shows strong interactions, that the destabilization of such adsorbates is important in determining activity. We thereby provide a quantitative understanding of the impact of absorbate interactions on volcano relations and the interpretation of the observed Tafel slopes for these electrocatalysts.

## Results and Discussion

### Physical and Spectroelectrochemical Characterization

CoOOH films were synthesized by the cathodic electroprecipitation method described by Boettcher and co-workers and operated in Fe free electrolytes (see Methods in the Supporting Information for details).^33^ Around 10 mC of charge was passed, and the resulting films exhibited a morphology consisting of multiple sheets (Figure S1a, scanning electron microscopy, SEM), consistent with previous findings, and were ca. 80–120 nm in thickness with a very high degree of roughness (Figure S1b,c, SEM, atomic force microscopy, AFM). X-ray photoelectron spectroscopy (XPS) confirmed the absence of Fe in the sample (Figure S1d). Due to insufficient thickness and the overlap of diffraction peaks with the flourine doped tin oxide (FTO) substrate, X-ray diffraction peaks could not be resolved; rather, the presence of the hydroxide/oxyhydroxide phase was determined using in situ surface enhanced Raman spectroscopy and X-ray absorption spectroscopy at a range of applied potentials (see Methods in the Supporting Information for details). Surface enhanced Raman spectra show a variation in Co–O breathing modes with applied potential (Figure S2a,b) consistent with previous in situ Raman studies of CoOOH by Bell as well as by Hu.^24,34^ X-ray absorption spectroscopy (XAS) shows similar K-edge positions (Figure S3a) and behavior to that reported by Boettcher, consistent with a similar material being formed.^35^ A small edge shift with increased applied potential was observed which has previously been attributed to Co^4+^ formation by Dau^15^ and Hu^24,36^ (but contested by Cheuh^27^).

Cobalt–iron prussian blue (CoFe-PB) films were synthesized by a previously reported hydrothermal method (see Methods in the Supporting Information for details).^37^ The resulting films were composed of cubic nanoparticles 100–700 nm in length (Figure S4a,b). The cubic structure and absence of bulk hydroxide phases was confirmed by X-ray diffraction (Figure S4c). XAS of CoFe-PB nanoparticles (see Methods in the Supporting Information for details) show Fe K-edge positions close to that of Fe(III), consistent with literature suggesting bulk oxidation states of Fe(III) being more stable in the bulk of the material (Figure S5a).^38^ Consistent with reports suggesting oxidation of both Fe and Co occurring under operation,^39^ a shift in both the Fe and Co K edges was observed to occur with increasing potential (Figure S5a,b).

Cyclic voltammograms of the two catalysts are compared in Figure 1c. Consistent with previous studies,^33^ CoOOH (measured in Fe-free KOH; see Methods in the Supporting Information, pH 13) shows a narrow, reversible redox wave occurring around 1.1 V_RHE_ with a slight asymmetry in the oxidation wave. The apparent onset of catalysis (defined here as the potential to observe 300 μA of current) is around 1.65 V_RHE_. In CoFe-PB (pH 7, phosphate buffer), a broader, more poorly defined set of redox waves begins at 1.1 V_RHE_ and extends into the OER region (onset at roughly 1.67 V_RHE_). We note that the CVs of CoOOH and CoFe-PB show marked differences in precatalytic redox waves regardless of the pH of the electrolyte (Figure S6). The CoFe-PB precatalytic redox wave is broader and more anodic than that of CoOOH across a range of pH from 7 to 12, indicating the distinct nature of these two materials under operation, regardless of pH. The pH most typically used for each material (13 for CoOOH and 7 for CoFe-PB) is used herein to maximize stability in CoFe-PB and for comparability to the literature.

**Figure 2:**
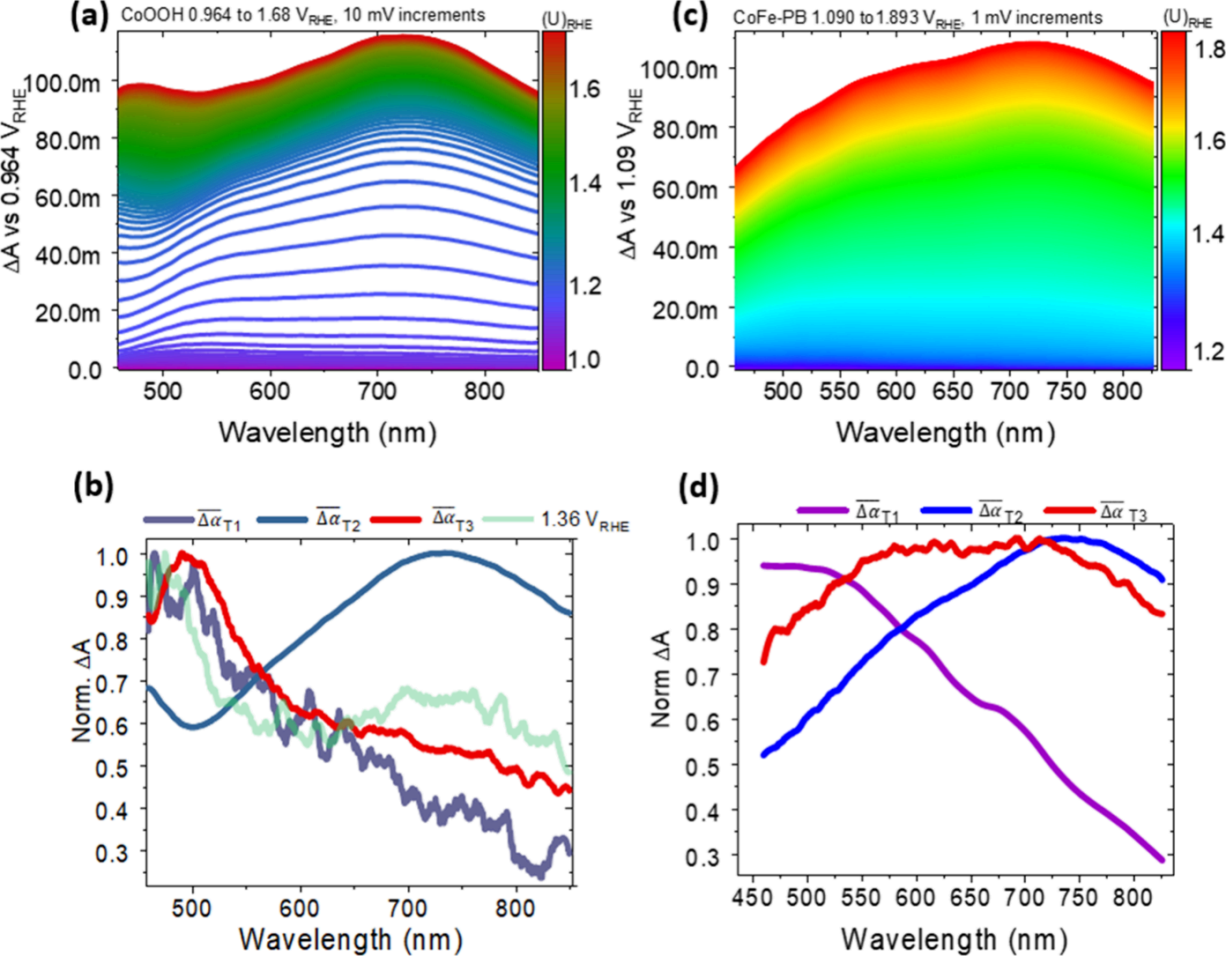
(a) Spectroelectrochemical differential absorbance spectra of CoOOH, measured with respect to the resting potential every 10 mV (corresponding to a scan rate of 10 mV s^–1^). (b) Normalized differential coulometric attenuation coefficient spectra of redox transitions in CoOOH. The normalized differential absorption shows the predominance of redox transition T3 even at cathodic potentials such as 1.36 V_RHE_. (c) Spectroelectrochemical differential absorbance spectra of CoFe-PB, measured with respect to the resting potential every 1 mV (corresponding to a scan rate of 1 mV s^–1^). (d) Normalized differential coulometric attenuation coefficient spectra of redox transitions in CoFe-PB.

To elucidate the populations of distinct oxidized states that give rise to the precatalytic waves and drive the catalytic current, spectroelectrochemistry was performed (see Methods in the Supporting Information for details). The change in optical absorption of CoOOH during the forward scan of a polarization curve, measured with respect to the absorption at the resting potential of the system (0.964 V_RHE_), is shown in Figure 2a. This shows a complex evolution of the absorption difference spectrum as a function of the potential. From the resting potential, a small initial absorption is observed toward the blue; this then gives way to a broad spectrum with a maximum around 750 nm at potentials around the precatalytic wave (ca. 1.1–1.2 V_RHE_). Following this, the absorption again grows toward the blue as the potential tends toward the onset of the OER. Complex spectral evolution can be analyzed by considering the normalized differential absorbance arising from a small potential increment (Figure S7a, see accompanying description for details) as this gives spectral fingerprints of the distinct redox transitions occurring in each potential step. The basic premise of the approach comes from the Beer–Lambert law, which states that the differential absorption spectrum arising from the electrochemical interconversion of two moieties (hereafter called a redox transition) will have a constant ratio of the absorbance at any two wavelengths regardless of the extent of reaction. Each differential spectrum therefore describes a distinct surface redox reaction. The total spectrum measured at each wavelength and potential is given by
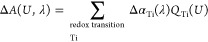
where Δα_Ti_(λ) is the differential coulometric attenuation coefficient of the *i*^th^ redox transition, with units of absorbance, area, per coulomb (the differential absorbance that would arise from passing 1 C cm^–2^ to drive a given redox transition) and *Q*_Ti_(*U*) is the partial charge that has been passed at potential (*U*) for a given redox transition (assuming a single electron couple). Please see Section S7 for a mathematical derivation of this equation and the equations for subsequent analysis procedures. In a potential region where only one redox transition takes place, taking the difference of two absorbances (Δ*A*(*U*_2_,λ) – Δ*A*(*U*_1_,λ)) and normalizing by the maximum (hereafter, the normalized differential increment spectrum) yields the differential coulometric attenuation coefficient normalized by its maximum value (see Section S7 for a derivation):
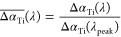
where Δα_Ti_(λ_peak_) is Δα_Ti_ evaluated at its peak wavelength. Thus, a region of applied potential where normalized differential increment spectra are identical can be assigned to a single redox transition (see discussion and equations accompanying Section S7). Three such regions are observed in CoOOH (Figure S7a) producing three unmixed differential increment spectra, corresponding to three normalized differential coulometric attenuation coefficients (Figure 2b) for the three distinct redox transitions in CoOOH:

- A very small initial differential absorption (hereafter redox transition 1, T1) whose differential spectrum slopes upward to the blue with a maximum at ca. 500 nm dominating from 0.9 _VRHE_ to around 1.05 V_RHE_. This can be seen to dominate in the purple region of Figure 2a and is shown in purple in Figure 2b.
- A large component with clear maximum around 750 nm (hereafter redox transition 2, T2), dominating from 1.1 to 1.3 V_RHE_. This dominates in the blue region of Figure 2a and is shown in blue in Figure 2b.
- Finally, a component with a maximum at 500 nm is observed (redox transition 3, T3) above 1.3 V_RHE_, discernible in the green/red regions of Figure 2a and is shown in red in Figure 2b.

Importantly, the switch from a differential spectrum dominated by redox transition T2 to one dominated by T3 occurs at potentials far below (0.4 V) the OER onset, with T3 dominating by 1.36 V_RHE_ (compare light green line and red line in Figure 2b; see also Figure S7a for onset of change at 1.2 V_RHE_).

Equally complex difference spectra are observed in CoFe-PB (Figure 2c), which can again be resolved into three differential coulometric attenuation spectra (Figure 2d, again named redox transitions T1–T3) by analyzing differential increment spectra (Figure S7b).

To decompose the data into partial charge, we perform a fitting procedure using normalized differential coulometric attenuation coefficient spectra. Combining the equations above gives
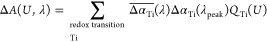


**Figure 3:**
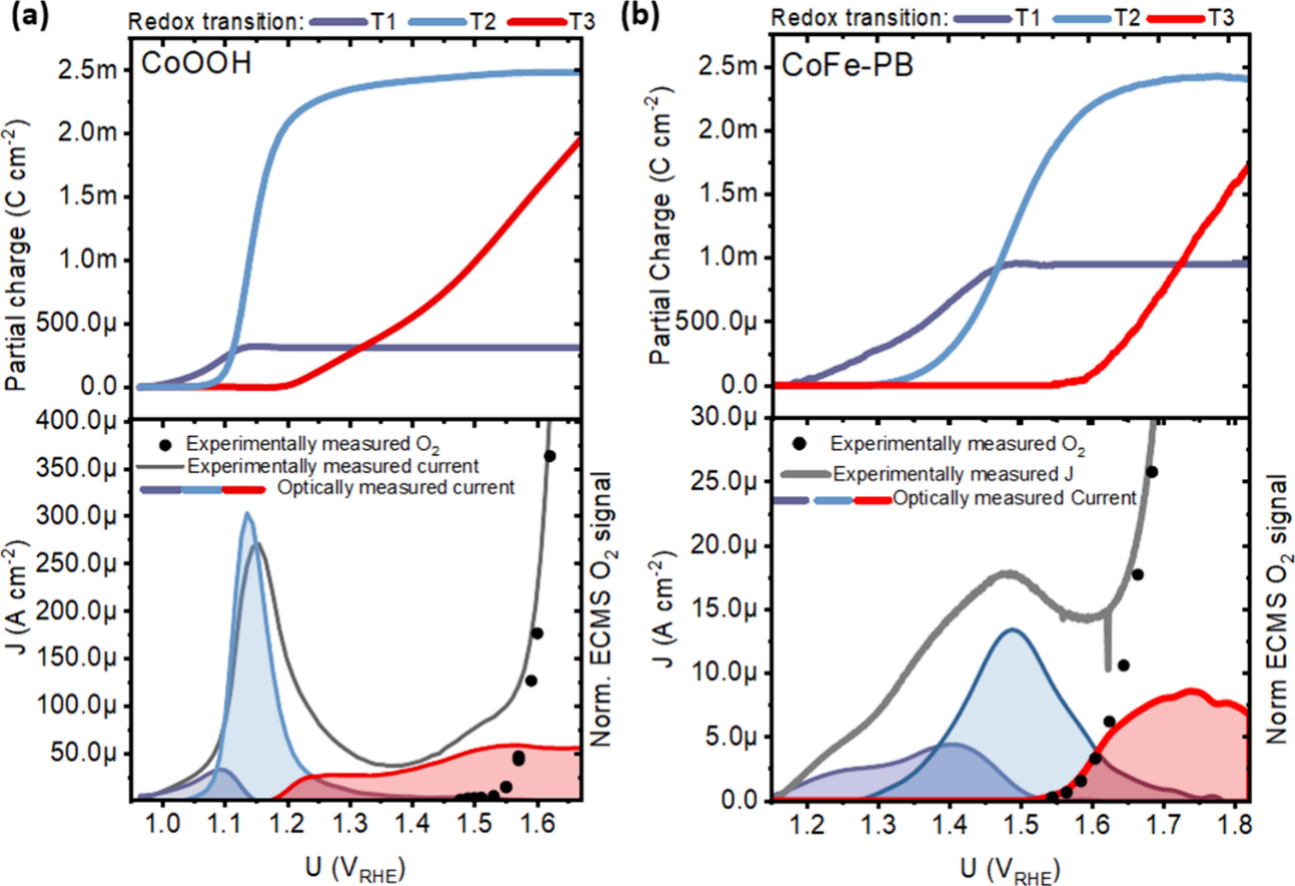
Top: The partial charge associated with each redox transition for (a) CoOOH (pH 13) and (b) CoFe-PB (pH 7). Bottom: Current predicted from the rate of change (capacitance) of partial charge multiplied by the scan rate for (a) CoOOH and (b) CoFe-PB. The gray line shows the current measuring simultaneously by the potentiostat during the same measurement. Black dots show the result of in situ EC-MS measurements performed on the same catalysts mounted on glassy carbon. The scan rate used in CoOOH was 10 mV/s while a 1 mV/s scan rate was used for CoFe-PB.

Fitting the data in Figure 2a,c to a linear combination of normalized differential coulometric attenuation spectra (Figure 2b,d; see Section S7 for details) allows the spectral evolution as a function of potential to be unmixed into contributions of distinct redox transitions (Δα_Ti_(λ_peak_)*Q*_Ti_). By dividing these values by empirically determined differential coulometric attenuation coefficients (Δα_Ti_(λ_peak_); Figure S8a–f; see accompanying description in Section S8 and Figure S7 for details), the partial charge associated with each redox process (*Q*_Ti_(*U*)) can be obtained.^16,18,19,21^ This is shown in the top panel of Figure 3a for CoOOH and the top panel of Figure 3b for CoFe-PB. Both materials exhibit an initial process, redox transition T1, associated with a smaller partial charge upon completion than subsequent ones. Thus, we tentatively assign T1 to a redox change associated with the oxidation of defect states, as this transition is more pronounced for CoFe-PB and consistent with its defective nature. Subsequent transitions T2 and T3 appear to show a commensurate partial charge. We therefore assign these processes to subsequent, stoichiometrically linked oxidations with a 1:1 ratio on the basis of this similarity and results from density functional theory calculations (see the following DFT for justification).

The top panel of Figure 3a gives a partial charge associated with each redox transition. Thus, the potential derivative of this result (i.e., faradaic capacitance), multiplied by the scan rate, should reproduce the faradaic charging current of the polarization curve. This is shown in the bottom panel of Figure 3a for CoOOH and the bottom panel of Figure 3b for CoFe-PB, which breaks down the charging currents predicted from our optical data into partial currents from the three distinct redox transitions. A strong match, prior to the onset of water oxidation catalysis, between the potentiostatic current measurement and optically predicted current demonstrates the validity of our approach and, for the first time, gives a complete and quantitative account of distinct chemical transitions present in the redox waves of CoOOH and CoFe-PB, as the sum of partial currents in the redox waves accounts for nearly all faradaic charging current. The precatalytic current cannot be accounted for by one redox process in either material, indicating that our measurements are able to access the inherent complexity of multiredox processes in electrocatalytic materials. In CoOOH, the calculated charging current attributed to redox transition T3 is drawn at significantly less positive potentials than the onset of OER (transition T3 current being drawn at ca. 1.2 V_RHE_ in Figure 3a). This is visible from clear changes in the differential absorption spectrum at this potential (Figure S7a; see also Figure 1e at 1.36 V_RHE_). This observation contrasts with the growth of redox transition T3 in CoFe-PB, which coincides with the apparent onset of the OER current.

### Time Resolved Open Circuit Decay to Assess the Activity of Intermediates

To unambiguously assign the current in the OER region to the turnover of states generated by T3, we separately performed in situ electrochemical mass spectroscopy (EC-MS; see Methods in the Supporting Information for details). The normalized rate of oxygen generation (fragment mass/charge, *m*/*z* = 32, black dots in lower panel of Figure 3) correlates with the generation of states produced by T3 in CoFe-PB but not in CoOOH. This result confirms that T3 product states are directly correlated with the OER in CoFe-PB but not in CoOOH. To address the possible interpretations of this lack of correlation, we turn to an analysis of the reaction kinetics of T3 product states in CoOOH, in order to determine the plausibility of the intermediate generated by T3 being the rate limiting intermediate for the OER.

**Figure 4:**
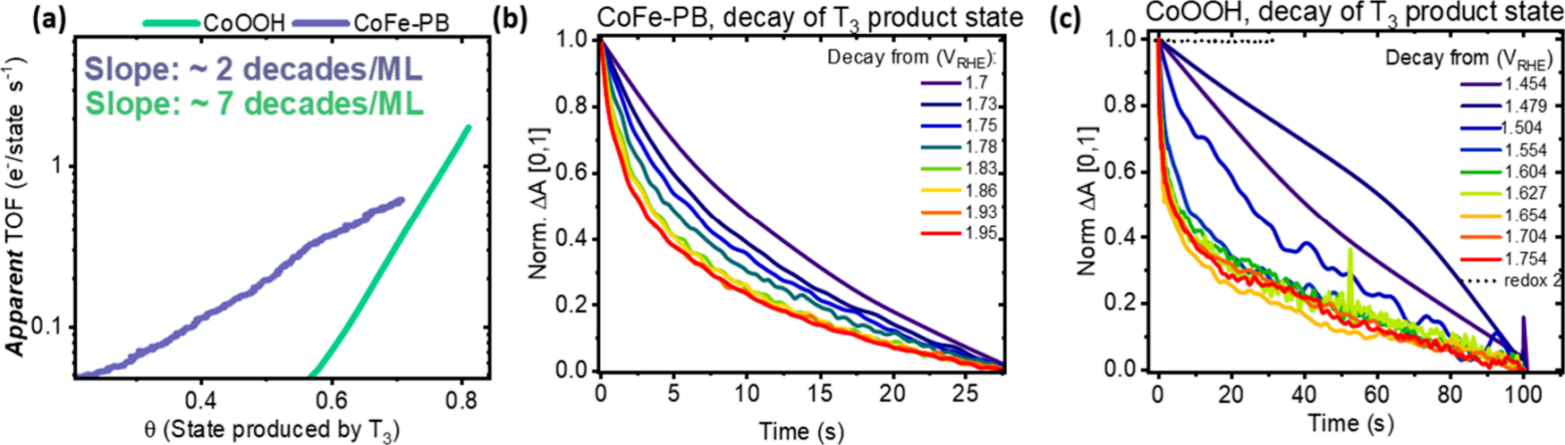
(a) Comparison of apparent TOF versus coverage of redox 3 for CoOOH and CoFe-PB. Here, we use the term **apparent** to imply that the states involved are assumed at this point, not yet proven, to be active for the OER. The activity of these states is demonstrated in (b) and (c) which show the normalized decay of redox 3 species as a function of time in CoFe-PB and CoOOH, respectively, at a series of increasing potentials corresponding roughly to the coverage range plotted in (a).

The identification of an oxidized intermediate does not necessarily indicate that the states measured turn over sufficiently quickly to account for the measured current.^22^ The states generated by redox transition T3 are not correlated directly with the onset of the OER in CoOOH. These states (i) do not participate in the OER, (ii) turn over to form the O_2_, but too slowly to account for the measured current, or (iii) must increase in activity with increasing coverage in order to drive the measured OER current. To distinguish these hypotheses, we begin by using the data in Figure 3 to calculate an ***apparent*** turnover frequency of the states produced by T3 (TOF, calculated in units of electrons (of current) per state (as stored charge) per second, related to O_2_ turnover by division by a factor of 4e^–^/molecule O_2_, assuming only one of the states produced by T3 is involved in the RDS), as a function of coverage, θ. Coverage is estimated as the ratio of the charge stored by redox transition T3 to the maximum produced by T2 (see the following DFT for a justification of this estimation of coverage). The resultant apparent TOF is plotted in Figure 4a for both CoOOH and CoFe-PB and will be compared below to direct kinetic measurements. We note that in both materials the logarithm of TOF scales linearly with coverage. This indicates an exponential dependence of the TOF on coverage. In the case of CoFe-PB, the slope of the linearized (log–linear) exponential plot is significantly less than that of CoOOH (2 vs 7 decades per monolayer); this indicates that the *apparent TOF* of the states driving OER in CoFe-PB is significantly less sensitive to coverage than in CoOOH. We also note with interest that this exponential dependence on coverage departs from the power law (i.e., rate law) behavior previously observed on metal oxide photoelectrodes and some nickel oxide/hydroxide electrodes.^16,40−43^ Power law behavior indicates a purely chemical RDS while the exponential dependence herein is consistent with either an electrochemical RDS or a chemical RDS, driven by interactions. It has recently been demonstrated that differences in power law versus exponential behavior between materials may result from the BEP transfer coefficient, which scales interaction energies with activation energy.^44^

To test the validity of the *apparent* TOFs, we time resolve the decay of spectra corresponding to the product of redox transition T3, to its starting state, as the electrode is switched from an applied potential to an open circuit. The product states of T3 were generated by applying a potential step in a region where little or no of the T2 process occurs to ensure that the differential absorption generated is attributable only to the T3 process. This was confirmed by the normalized differential spectra resulting from the open circuit decays matching the pure T3 spectrum for both samples (Figure S9a,b). Figure 4b,c shows the normalized decay kinetics of the product state of T3 under open circuit conditions of CoFe-PB and CoOOH from a series of increasing applied potentials. For CoFe-PB, the time scale of the initial rate of decay accelerates from ca. −360 μOD/s to ca. −7 mOD/s as the potential is increased above the onset of catalysis. This corresponds to an ∼8-fold increase in TOF with increased coverage (ca. 0.04 e^–^/state s^–1^ to 0.32 e^–^/state s^–1^, Figure S10a,b), commensurate to the increase in TOF observed in Figure 4a. In CoOOH, a more dramatic acceleration in the decay kinetics is observed. At potentials before the onset of O_2_ evolution (<1.5 V_RHE_), the initial rate of the optical signal at 500 nm decays at −40 μOD s^–1^. Due to a larger charge stored as T3 (TOF = current/charge; see discussion in Section S8 for details) in CoOOH, arising from the early onset of T3, this corresponds to a TOF of 0.004 e^–^/state s^–1^. Despite this, as the onset potential of the OER is exceeded, the decay rate reaches −9 mOD s^–1^, corresponding to an acceleration of the initial rate of decay by more than 2 orders of magnitude to 0.54 e^–^/state s^–1^ (Figure S10c,d). The TOFs as a function of coverage calculated from SEC and from decay kinetics agree (Figure S10c,d). Such a match indicates that the turnover frequency measured by the initial rate of decay of states generated by T3 is sufficient to explain the OER measured in both CoFe-PB and CoOOH, implicating the states generated in the T3 process as the catalytic intermediate driving the OER and accumulating before the RDS, and confirms the apparent TOFs in Figure 4a as an accurate estimation. This correlation provides a complete account of the activity of the distinct states produced by different redox processes in CoOOH, as the state produced by T2 does not decay (black dots, Figure 4c). This indicates that T2 product states are inactive, and activity is attributable to the accumulation of the states produced by redox transition T3.

Dividing the upper value for CoOOH (0.54 e^–^/state s^–1^ at 1.754 V_RHE_) by 4e^–^/molecule O_2_ gives a TOF of 0.135 O_2_ molecules/state s^–1^ and enables comparison to the literature values. Agreement is obtained with the TOFs reported by Burke et al. across a wide range of potentials, made using accurate measurements of the number of cobalt atoms deposited (Figure S10e).^45^ This would suggest that in the case where films are deposited in such a way as to maximize the number of available active sites the number of optically/electrochemically active sites and the total number of sites become similar, leading to similar TOF values.

### Experimental Estimates of Interaction Energy

**Figure 5:**
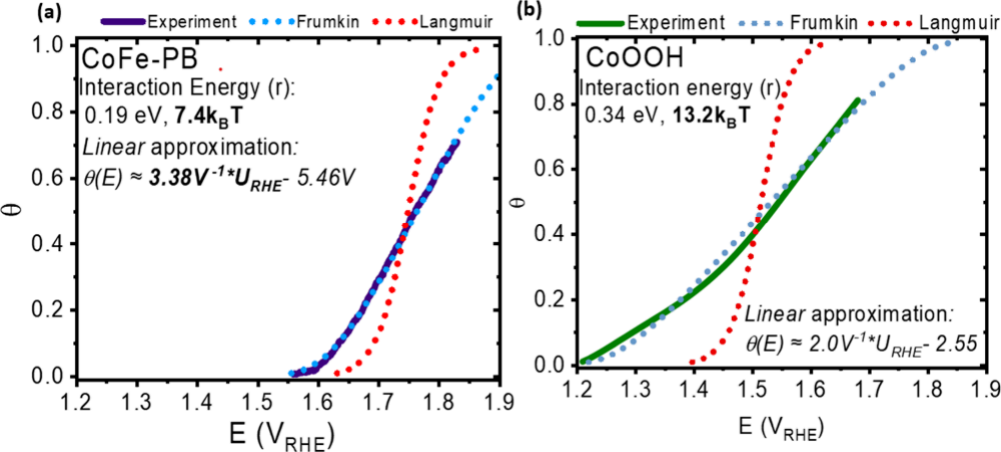
Coverage of redox transition T3 as a function of the potential for CoFe-PB (a) and CoOOH (b). This is fitted to two electroadsorption models. In both cases, a simple Langmuir model (, red dots) cannot account for the data, whereas a good fit is obtained for a Frumkin model (, blue dots). For the Frumkin model, the fitted interaction energy is also given alongside the simplified form of the isotherm, which describes the linear region of the isotherm termed “linear approximation”.

Given kinetic evidence that redox transition T3 in CoOOH can be assigned as a catalytic intermediate, we turn now to examining the physical origin of the exponential acceleration of turnover frequency with population. If the acceleration in rate arises from destabilization of rate limiting intermediates, this effect must manifest itself in the thermodynamics governing the generation of the intermediate, namely, as a “stretching” of the electroadsorption isotherms as species become harder to oxidize with increased coverage. Figure 5a shows the coverage of the product of redox transition T3 in both materials as a function of the applied potential. Two electroadsorption isotherm models were used to fit the data:

- The Langmuir model,
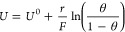
where the enthalpy of adsorption is assumed to be independent of coverage, leading to a Nernst-like dependence and a characteristic standard potential at  when .
- The Frumkin model,
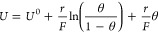
where an additional term is added to describe destabilization with increased coverage.^4^ The linear term in the Frumkin isotherm  could describe the disruption of long-range interactions with increasing coverage, which in turn drives or helps to drive the rate limiting step. In the Frumkin model, the binding potential of the rate limiting intermediate is itself a function of the coverage (where *r* is an interaction energy and *F* is Faraday’s constant), and  when .^6^

For CoFe-PB, the evolution of the product of redox transition T3 with potential cannot be described by a Langmuir model (Figure 5a; see Figure S11a, top, for full fitting, parameters, and *R*^2^). However, a good fit to a Frumkin isotherm with an interaction parameter (*r*) of 0.19 eV per monolayer is observed. Frumkin electroadsorption implies that the enthalpy of catalytic intermediates increases as a function of potential as bonding between intermediates is destabilized.^6^ CoOOH showed a very poor fit to a Langmuir model, and a good fit to a Frumkin isotherm is observed (Figure 5b, for full fitting, parameters, and *R*^2^; see Figure S11a, bottom. See also Figure S11b for fits used in subsequent kinetic modeling). In this case, a much larger interaction of 0.34 eV/monolayer is measured. These results indicate that the thermodynamics of the catalytic redox transitions are strongly affected by interactions in both materials.

### Assigning Optically Observed Redox Transitions to Surface Redox Processes

**Figure 6:**
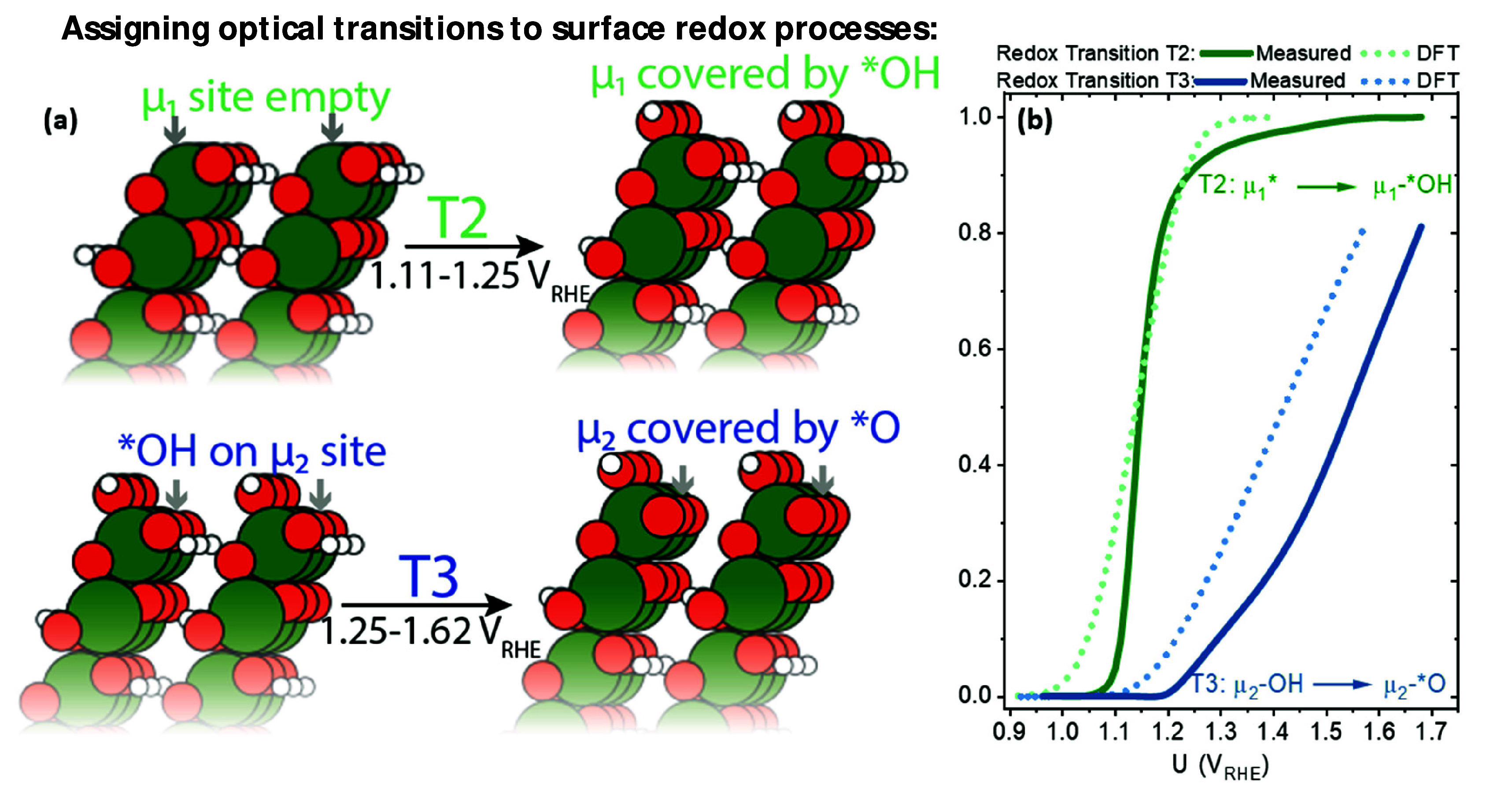
(a) Assignments of T2 and T3 in CoOOH. Top: The lowest calculated potential of oxidation of cobalt on a {0112} termination of CoOOH involves adsorption of OH on a coordinatively unsaturated μ_1_ site. Bottom: This is followed by the oxidation of μ_2_-OH sites on the same terminal Co atom. (b) The experimental coverage dependence of T2 and T3 shows excellent correspondence with the calculated lowest potential redox transitions, and the assignment is made accordingly.

To (1) validate empirically observed Frumkin isotherms and (2) assign the states that interconvert during redox transitions T2 and T3 in CoOOH to specific surface redox processes, we turn to density functional theory (DFT) calculations. Repeated oxidation of the same site type has previously been shown to become more energetically challenging with increased coverage in iridium-based catalysts as a result of adsorbate interactions.^6^ To include the possibility of this phenomenon, we model the effect of repeated proton coupled oxidations of identical sites in an extended unit cell. This procedure was performed on several different β-CoOOH surfaces: the {0001} termination, running parallel to the basal plane of the sheets, and the {0112} and {1014} terminations, both of which intersect the basal plane to form sheet edges. These two edge terminations differ in the angle of the sheets relative to the surface plane, but both terminate in a 1:1 mixture of surface μ_1_ and μ_2_ sites (Figure 6a, arrows on top and bottom panels), albeit in differing relative locations. All three surfaces are modeled by slabs with five layers of Co atoms, with the bottom two fixed in the position of the bulk atoms. The surface termination at different potentials is probed by considering the full coverage of *OH, *O, and *H_2_O on both the coordinatively unsaturated (μ_1_-*) and μ_2_-*OH sites at the stoichiometric surfaces of the edge terminated (0112) and (1014) surfaces, as well as deprotonation of the *OH groups residing on the μ_3_-sites on the basal plane of the (0001) surface. In the case of the {0001} termination, we find that for the lowest energy surface at relevant potential for OER μ_3_-sites are already deprotonated from μ_3_-*OH to μ_3_-*O (Figure S12a) and that OER has a significant overpotential (1.43 V overpotential needed, Figure S12b). This agrees with previous theoretical^23^ and experimental^27^ observations suggesting that OER happens primarily at terminations of the basal plane, i.e., at the edges of a macroscopic crystal or at steps between terraces, and enables us to discount this surface as being associated with T3 in CoOOH.^27^

Both the {0112} and {1014} terminations are found to be active for OER at experimentally relevant overpotentials (e.g., 1.6 V_RHE_), and both show broadly commensurate behavior, which is summarized for the {0112} termination in Figure 6a. At potentials below 1.12 V_RHE_, we find {0112} surface μ_2_ sites to be occupied by doubly bridging *OH (i.e., μ_2_-OH) and coordinatively unsaturated cobalt at the μ_1_ sites (i.e., μ_1_-*, Figure 6a top; Figure S13a). At potentials above 1.12 V_RHE_, OH^–^ is adsorbed on μ_1_ sites to form terminal μ_1_-OH (Figure 6a, top). After the first adsorption/oxidation, neighboring sites become harder to oxidize, leading to subsequent oxidation potentials for three neighboring μ_1_ sites along the periodically repeated sheet edge of 1.12, 1.14, and 1.25 V_RHE_. Positive of 1.25 V_RHE_, the oxidation of terminal μ_2_-*OH sites to μ_2_-*O was found to occur (Figure 6a, bottom; Figure S13a). Here, significantly more destabilization with increasing coverage occurs, with calculated oxidation potentials for the three sites at 1.25, 1.42, and 1.62 V_RHE_. By taking the difference between the first and last oxidation of each process as an estimate of the Frumkin interaction parameter (*r*), the potential of the central oxidation process (i.e., *E*_1/2_) can be calculated and used to produce a theoretical adsorption isotherm (see Table S1 and discussion accompanying Figure S13 for details).^4^

A comparison of the DFT simulated coverages for CoOOH versus those determined experimentally is shown in Figure 6b. Good agreement between theory and experiment is obtained for redox transitions T2 and T3 (reproduction of *r* and *U*_1/2_ to within 100 mV error; see Table S1 and accompanying discussion for numeric comparison). Based on this match, we assign the optical redox transition T2 as the electroadsorption of hydroxide on μ_1_ sites (OH^–^ + μ_1_-* → μ_1_-*OH + e^–^) and T3 to the oxidation of μ_2_-OH sites to μ_2_-O (OH^–^ + μ_2_-*OH→ μ_2_-*O + e^–^ + H_2_O). The 1:1 stoichiometry of μ_1_-OH adsorption to μ_2_-OH oxidation matches the commensurate concentrations of the processes observed in Figure 3 and justifies our calculation of θ as the ratio of the density of the states produced by redox transition T3 to the maximum density produced by redox transition T2, as μ_1_ and μ_2_ oxygens form on terminal cobalt atoms in a 1:1 ratio. Similar attenuations of intermediate energy as a result of changing coverage can be observed in the {10114} surface, which intersects the basal plane from a different direction and at a shallow angle (Figure S14a–c and Table S2 for details). These results provide a degree of confidence that the observed effects are not limited to a single type of abrupt termination of the basal plane and are applicable to the highly stepped and disordered morphologies studied herein.

The assignment of redox transitions T2 and T3 to the oxidation of a terminal site and a bridge site, respectively, broadly agree with literature assignments of the first two steps in the proposed catalytic cycle producing a cluster containing a terminal oxo species and bridging oxo species Co(μ_1_*OH*)–*O*–Co(μ_1_*OH*) at lattice terminations, with the oxidation states being either 4+ 4+^24^ or 4+ 3+.^15^ In contrast to Moysiadou,^24^ we do not find evidence (Figure S2) of a superoxo cluster (Co^3+^(μ_1_*OH*)–*O*–*O*–Co^3+^(μ_1_*OH*)) forming in equilibrium from adjacent bridging oxygens in the cobalt to the cobalt oxo cluster. We therefore tentatively suggest the rate limiting step does not involve the desorption of this intermediate but rather an O–O bond forming step involving the nucleophilic attack of water or OH^–^ on the cobalt oxo cluster to form a peroxo intermediate.

The catalytic sites in the CoFe-PB, at least in the crystalline structure, are significantly further separated (>1 nm) when compared to the stacked sheet structure of CoOOH. It is plausible then to expect a lower degree of interaction based on the separation of cobalt species. An indication of this can be obtained by performing similar simulations on CoFe-PB by analyzing the Frumkin parameter for *OH and *O formation on defective CoFe-PB surfaces produced by introducing Fe(CN)_6_ vacancies (Figures S15 and S16 and Tables S3–S6 for details and discussion). In agreement with this hypothesis, lower interaction parameters are obtained. However, the complexity and heavily defective nature of the PB network prevents us from accurately simulating this effect. We therefore restrict detailed analysis to CoOOH herein and use CoFe-PB as another catalyst to contrast our results to, in which the effect of interaction is less strong.

### A Simple BEP Model Connecting the Rate of Reaction to Coverage

Our results suggest that the characteristic binding energy of the rate limiting intermediates in CoOOH and CoFe-PB is not constant but rather varies linearly with coverage:
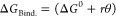
Approximately linear correlations between activation energy and free energy (i.e., BEP relations) are well established in gas phase catalysis.^5^ Suggestions that charging an electrode influencing rate though the destabilization of states via a BEP relation date back to measurements made by Conway in the late 50s.^12^ However, the last 20 years have generated theoretical evidence for the validity of BEP in electrocatalysis.^4,6,44,46−48^ On this basis, we explore the hypothesis that BEP scaling influences rate:
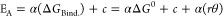


By combining constants, we simplify to
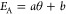


**Figure 7:**
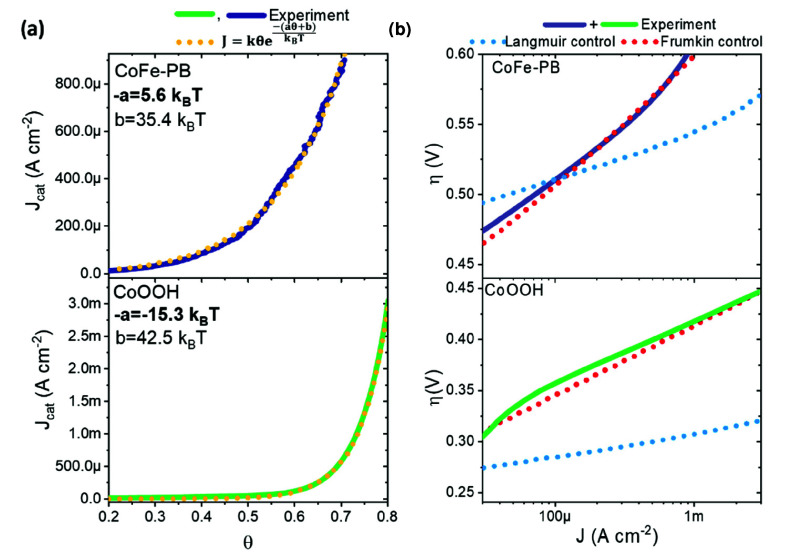
(a) A simple first order, exponential product fit of current as a function of coverage for CoFe-PB (top) and CoOOH (bottom); here, the slope in Figure 3a takes on the meaning of the fitting parameter *a*, the BEP scaling coefficient. (b) Experimental Tafel slopes for CoFe-PB (top) and CoOOH (bottom). These are compared to the Tafel slope obtained by taking the dependence of coverage on potential from the linear relation and the Langmuir equation from Figure 5 and substituting for coverage in the rate equation given by the yellow dots in (a); see Section S11 for fitting details. This indicates that Tafel slopes are strongly affected by the effect of absorbate interactions on coverage.

To further interrogate the hypothesis that destabilization drives current increase in both materials, we begin by following the work of Nong et al.,^6^ fitting the coverage dependence of catalytic currents to a first-order kinetic equation, where activation energy is modulated by BEP scaling with the free energy of the rate limiting intermediate with increased coverage:
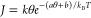
In this formalism, the first parameter, *a*, represents the coverage dependent BEP scaling coefficient, the degree to which *E*_a_ is diminished by increasing coverage. The second parameter, *b*, in principle represents the activation energy at zero coverage. The prefactor, *k*, is not a fitting parameter, but rather is calculated according to a formulation of the Eyring equation modified by Nong et al.^6^ for OER, ,^11^ where *q*_e_ is the elementary charge in coulombs, *k*_B_*T* is the thermal energy in eV, *h* is Planck’s constant in units of eVs, and *N* is the number of available sites per cm^2^, which we estimate from the maximum number of states produced by T2 in Figure 3a,b, top (see Figure S11b for fitting parameters and accompanying discussion for details; partial charge of T2 is converted to states using Faraday’s constant). An excellent fit is obtained for both materials (Figure 7a), reflecting the fact the logarithm of current is linearly dependent on coverage (e.g., Figure 4a) but not linearly dependent on potential as reflected in the gently curving Tafel slopes reported in the literature.^24,49^ We note that the physical origin of this model is distinct from Butler–Volmer-like formalisms, as here, the initial barrier height is accounted for in the exponential term rather than pre-exponential term. Thus, although *k* has units of A cm^–2^, it should not be interpreted as an exchange current density, but rather as an attempt frequency scaled by the number of available active states (*N*·θ) and by the charge that would be transferred by the reaction (4e). The effect of the *k* parameter is to effectively scale *b* during the fitting. As direct activation energy measurements are beyond the scope of this work, we do not therefore confidently interpret *b* as an activation energy without independent validation of this value, as errors in our data or further refinement of the equation describing *k* will influence the fitted value of *b*. Unlike Nong et al.,^6^ we do not rule out an electron transfer occurring in the RDS; however, given the destabilization of intermediates, it is likely that BEP scaling significantly contributes to current.

Further to this point, we report with interest that the interaction energy (*r*), obtained from the Frumkin isotherm for both catalysts (*r* = +7.4 *k*_B_*T* and +12.2 *k*_B_*T* per monolayer for CoFe-PB and CoOOH, respectively), is commensurate to the change in activation energy predicted by the BEP rate model (*a* = −5.2 and −15.6 *k*_B_*T* per monolayer of rate limiting intermediates for CoFe-PB and CoOOH, respectively; see Figure 7a and following discussion).^44^ If such a correspondence is not merely coincidental, it would imply a late transition state and a BEP transfer coefficient (α_BEP_ connecting intermediate free energy to activation energy attenuation, α_BEP_·*r* = −*a*) approaching unity. Such a conclusion is beyond the scope of this work but hints that, consistent with studies of iridium oxides,^6^ the destabilization of rate limiting intermediates with increased coverage may predominantly contribute to the measured potential dependence of the OER rate, especially in the low overpotential regime. Herein, we conclude that it is reasonable to assume that the BEP transfer coefficient is nonzero, indicating that cooperative effects drive water oxidation catalysis in cobalt electrocatalysts through the destabilization of intermediates. A near unity value of the BEP transfer coefficient may distinguish the materials studied herein from the behavior exemplified by Fe_2_O_3_ photoanodes, which shows simple third order chemical reaction kinetics. This difference has been proposed to arise from a late transition state, in which the BEP transfer coefficient is on the order of 0.1.^44^

The destabilization driving the OER can also be explained in terms of the optimality of binding energy of intermediates. The experimental value of *U*^0^ for the formation of the OER active μ_2_-*O site (1.37 V_RHE_) in CoOOH indicates binding which is too strong to exhibit optimal OER kinetics.^50^ Destabilization with increased coverage would therefore lead to a lower activation energy as a result of BEP scaling (i.e., weakening of binding with increased coverage, leading to a linear decrease in activation energy).^6^ The agreement between predicted and measured isotherms suggests that cooperative effects, arising from the strong (∼0.34 eV/ML) destabilization of μ_2_-*O species as transition T3 proceeds, help drive the acceleration in the rate of OER in CoOOH.^6^ We suggest this strong interaction transmitted as bonding changes on μ_2_-sites because Co atoms are shared by neighboring bridging oxygens along the sheet edge (i.e., the oxidation of μ_2_-*OH requires more energy if a neighboring site is oxidized to μ_2_-*O). This effect is visible in the energies of the calculated intermediates of the OER, which are strongly modulated by the cumulative effect of this coverage dependent destabilization, regardless of the potential chosen (Figure S12b–d). This strong interaction may account for the offset between the generation of the product state of T3 and oxygen evolution. In contrast, CoFe-PB shows a more weakly bound intermediate (*U*^0^ = 1.68 V_RHE_) with a destabilization (0.19 eV/ML) roughly half that of CoOOH, possibly due to the separation of cobalt sites in the ideal lattice that may template in any induced defect clusters and overlayers.

### Effects of Frumkin Behavior on the Interpretation of Tafel Slopes

These interaction dominated adsorption equilibria are observed for two dissimilar catalysts, which has crucial consequences for the interpretation of the Tafel slopes. Although often criticized,^33,51^ a change in Tafel slope is commonly (i) suggested to arise from a mechanistic change in the rate limiting step^52^ or (ii) interpreted in the context of simple mechanistic microkinetic models, typically involving ideal electrochemical pre-equilibria forming catalytic intermediates which go on to react in a chemical rate-determining step.^53,24^ Consistent with the warning made by Nong et al. on IrO_*x*_,^6^ our data indicates that even in the case of CoFe-PB, where interactions are weaker, assumptions involving the Nernstian behavior of pre-equilibria and the rate limiting step employed in the interpretation of Tafel slopes will not be valid if the binding energy associated with such pre-equilibria and the activation energy contain linear functions of coverage. This can easily be shown for our data by taking the fitted exponential relationship between current and coverage (Figure 7a) and substituting the coverage for the implicit potential dependence from the Langmuir (typically used) and Frumkin (observed herein) relations. (Note: as no analytical solution exists for the θ in the Frumkin isotherm, the simplified linear relation, shown in Figure 5, was used). This allows the implicit potential dependence from coverage on the current and thus the Tafel slope to be explored. The resulting Tafel slopes derived from the linearized Frumkin relation (red dotted lines in Figure 7b) match well with our experimentally determined Tafel slopes (solid lines in Figure 7b) and agree well with the literature values (ca. 60 mV/dec for CoOOH^24,37^ and ca. 90 mV/dec for CoFe-PB^49^). From these results, we conclude that Langmuir electroadsorption, which is commonly assumed in the literature,^24^*cannot simultaneously account for the experimentally observed Tafel slope and the experimentally determined coverage*. Consequently, the Tafel slope cannot exclusively be attributed to (i) the rate-determining step or (ii) the number of pre-equilibria before the rate limiting step (assuming coverage independent rate constants in the rate limiting step) without direct measurements of coverage justifying these assumptions. Here, the interaction parameter in the pre-equilibrium affects not only how coverage changes with potential but also how this coverage changes the activation energy, both of which play a key role in determining the observed Tafel slopes.

## Conclusions

A fundamental understanding of the factors driving oxygen evolution catalysis is a crucial step in identifying the factors limiting the performance. In this work, we assess the role that adsorbate interactions play in two cobalt containing catalysts. In CoFe-PB, we observe weaker interactions and a direct correlation between the emergence of catalytic states from optical spectroscopy and the onset of oxygen evolution from mass electrochemistry mass spectrometry. This is consistent with more weakly bound states with interactions playing a smaller role in modulating the activity. However, in CoOOH, the formation of catalytic intermediates occurs at a significantly lower anodic potential than the onset of oxygen evolution. First-principles calculations and experimental electroadsorption isotherms show a strong destabilization of catalytic intermediates with increased coverage in CoOOH. This explains the generation of states at potentials less positive than the onset of O_2_ evolution, as this offset is associated with an ∼100-fold increase in turnover frequency increased coverage. We trace this phenomenon back to a 0.35 eV/monolayer destabilization of catalytic intermediates, suggesting that interactions play a crucial role in determining the activity and Tafel slope of CoOOH. Crucially, however, in CoFe-PB, where interactions are weaker, they still significantly broaden the electroadsorption isotherm and also appear to contribute to current. Such behavior is not accounted for in simple interpretations of Tafel slopes, where both the redox potential of pre-equilibria and the activation energy are assumed to be independent of coverage. Our results strongly indicate that the potential dependent interaction of intermediates is a non-negligible factor in controlling the kinetics of complex electrocatalysts. Such effects offer new horizons for better understanding and controlling the activation barriers of oxygen evolution electrocatalysts by (i) identifying the role adsorbate interactions play in dictating both the Tafel slope and dynamically positioning a material on the volcano curve and (ii) taking steps to control the strengths of interactions through careful surface and electrolyte modification.

## Data Availability

DFT optimized structures and scripts used to produce related figures can be obtained from https://nano.ku.dk/english/research/theoretical-electrocatalysis/katladb/oer-on-coooh-and-prussian-blue/. Optical spectroscopy data is available on Zenodo, https://zenodo.org/records/10727176. All other data will be made available on reasonable request.

## Supporting Information Available

Information on sample synthesis and characterization, the methods used for DFT and optical spectroscopy, derivations of the equations used to analyze the data, and additional optical spectroscopic measurements of the coulometric attenuation coefficient well as additional DFT calculations of binding on other surfaces of CoOOH and CoFe-PB (PDF)
