# Supplementary material for: Cooperative Effects Drive Water Oxidation Catalysis in Cobalt Electrocatalysts through the Destabilization of Intermediates
Source: J Am Chem Soc. 2024 Mar 22;146(13):8915–27. doi: 10.1021/jacs.3c11651 (PMC10995992; doi:10.1021/jacs.3c11651)
Supplement: Supplementary file 1 — ja3c11651_si_001.pdf [file ja3c11651_si_001.pdf]

**Supplementary information for Cooperative effects drive water oxidation catalysis in cobalt electrocatalysts through the destabilisation of intermediates.**

Benjamin Moss, Katrine Louise Svane, David Nieto-Castro, Reshma R Rao, Michael Sachs, Cindy Tseng, Anuj Pennathur, Soren B. Scott, Caiwu Liang, Louise I Oldham, Eva Mazzolini, Lole Jurado, Stephen Parry, Veronica Celorrio, Gopinathan Sankar, Jahan M Dawlaty, Jan Rossmeisl, J.R. Galán-Mascarós, Ifan E. L. Stephens, James R Durrant.

## Methods

### Electrochemistry

All samples were measured in a 3-electrode configuration employing an AgAgCl (Sat.d KCl) as reference, and an aqua regia cleaned Pt mesh as counter. Samples were compensated for the iR drop, calculated from the high frequency intercept of a Nyquist plot of an electrochemical impedance measurement at 1.55 $V_{RHE}$. All plots are made using the IUPAC convention for current and polarisation. A surface area of ~ 1 $cm^2$ and ~ 1.6 $cm^2$ was used to calculate current density of the CoOOH and CoFe-PB electrodes respectively. Samples were measured in a home built polyetheretherketone cell with no membrane or diaphragm separating the electrodes.

### Sample Preparation

CoOOH was deposited using a method based on the cathodic electroprecipitation procedure reported by Burke and Co-workers.[1] Here, -56 µA was passed for 180 s in a three-electrode configuration (using AgAgCl (Sat.d Cl) as a reference and carbon paper (Toray) as a counter, with rigorously cleaned FTO as a working electrode) through a solution of 10 mM solution of $CoNO_3$. The resulting films were washed in ultra-pure water and cycled three times in Fe free KOH (0.1 M) at 10 mV/s before use.

CoFe-PB on FTO was prepared using a previously reported hydrothermal method.[2] Briefly, CoOx was solvothermally deposited on FTO by heating $Co(NO_3)_2$ and carbamide in an autoclave for 3h at 120ºC. The sample was subsequently derivatised for 2h in hexacyanoferrate ($K_3[Fe(CN)_6]$, 12 mM) solution, to form CoFe-PB. Residual CoOx was removed by an acid treatment, i.e. immersing the electrodes for ca. 3 hours in diluted $H_2SO_4$ (pH = 1). Cubic crystals of around 300-600 nm are formed producing a purple/brown coloured electrode.

### Electrolyte purification

KOH electrolytes were cleaned according to the procedure by Boettcher et. Al.[3] Briefly, Merk SupraPur KOH was made into a 1M solution using 18 M Ohm deionised water. 50 mL electrolyte was added to 2 g of Cobalt nitrate (99.99 %, Sigma) to precipitate Cobalt hydroxide. The electrolyte was decanted and a three further washings with the KOH was performed. Finally, 50 mL of the KOH was added, sonicated in the precipitate, and then left for 2 hours. The suspension was then centrifuged and the clean electrolyte decanted and stored in an acid washed plastic bottle. This process was repeated until the desired quantity of electrolyte was obtained.

### SEM

SEM was performed on a ZEISS LEO GEMINI 1525 at an accelerating voltage of 2.5 keV and a working distance of 3 mm. Samples were measured on FTO substrates electrically connected to an SEM stub and coated in 10 nm of Cr before measuring.

### SERS

All spectra were taken with a 532 nm laser from a Raman instrument (Horiba Scientific) with a 100 x focusing objective. Spectra were taken every 5 minutes with a 1200 gr/mm grating with the sample placed in a custom built cell. Samples were deposited on a roughened gold substrate made according to Bell and co-workers.[4]

**EC-MS**

EC-MS was performed on a Spectro-inlets professional system using a custom designed electrochemistry cell in a 3-electrode configuration. Samples were deposited on a 5 mm diameter glassy carbon disc. A nanoperforated silicon ship was placed below the sample, allowing oxygen to diffuse into the carrier gas (He) flowing under vacuum. To clearly distinguish the potential at which oxygen was evolved a square wave voltammetry procedure was used with a starting potential of 1.23 $V_{RHE}$. The step size was iteratively increased in increments of ca. 20 mV and the upper potential was held for around 100 s to able $O_2$ produced by the reaction to diffuse into the carrier gas and pass though the mass spectrometer. The resulting M/Z=32 signal was

**XAS**

The Co and Fe K-edge XAS was performed B18 of the Diamond Light Source. Fe foil was used to calibrate the monochromator. XAS data for all the reference materials were collected using fluorescence mode in using a home built teflon cell, in which the sample was placed ~2 mm from a Kapton foil window.  The energy of the incident X-ray beam was selected using a Si (111) monochromator, and measurements were performed in fluorescence mode.

**XPS**

XPS was performed on a Thermo Scientific K-alpha+ instrument. Powdered samples were attached to a stainless-steel plate using conductive carbon tape. The instrument uses monochromated and microfocused Al Kα (hν = 1486.6 eV) radiation to eject photoelectrons which are then analysed using a 180° double-focusing hemispherical analyser with a 2D detector. Spectra were collected at $2x10^{-9}$ mbar base pressure. A flood gun was used to minimize sample charging. All samples were referenced against the C-C peak of adventitious carbon in the C 1s spectrum at a binding energy of 284.8 eV to correct for any charge that is not neutralised by the flood gun. Further effects were then accounted for by taking the separation from the O 1s oxide peak. Data was analysed using the CASA XPS package.

**XRD**

X-Ray diffraction (XRD) patterns were measured in the range 5º ≤ 2θ ≥ 70º, step size 0.02º, using a Bruker D2 Phaser instrument (Cu $K_\alpha$ radiation source).

**SEC**

Spectroelectrochemistry was performed using a stabilized 10mW tungsten-halogen light source from Thorlabs (SLS201L) was used with a collimating add on (SLS201C). The light emitted from the lamp was transmitted through the sample and collected using a 1 cm diameter liquid light guide (Edmund optics). Light transmitted to the spectrograph was first columnated and refocused using two 5 cm planoconvex lenses (Edmund) in order to optimally match the optical components of the spectroscope (Kymera 193i, Andor), CCD camera (iDus Du420A-BEX2-DD, Andor). The detector was maintained at -80$^o$C during the measurements to ensure high signal-to-noise ratio. An Ivium Vertex potentiostat was used. Data acquisition was facilitated by a custom-built LabView software, freely available at www.opensourcespectroscopy.com. Measurements were made in potentiostatic mode using a Pt mesh as counter and a AgAgCl (Sat.d KCl) as reference. Samples were measured in a homemade

polyether ether ketone (PEEK) cell, were light is light transmitted through the sample via two quartz windows.

Unlike typical spectroelectrochemistry, samples were scanned continuously thought their operational JV curve at a set scan rate. The change in absorbance ($\Delta A$) was calculated with respect to the starting potential of the scan according to:

$$\Delta A = -\log\left(\frac{I_V}{I_{V0}}\right)$$

Where $I_V$ is the intensity of transmitted light at some measured potential (after iR correction) and $I_{V0}$ is the intensity of transmitted light at the potential (i.e. at the start of the scan). The MATLAB code for converting raw data to absorbance (SEC_V_3_2_KYMERA_iR.m) is freely available at www.opensourcespectroscopy.com

**DFT**

Spin-polarised density functional theory (DFT) calculations were performed with the Vienna Ab-initio Simulation Package (VASP),[7] and the Atomic Simulation Environment (ASE) was used to set up and analyse the calculations.[8] A plane-wave basis with an energy cutoff of 700 eV was used, and the effects of exchange and correlation were described with the RPBE functional.[9]

The oxygen evolution reaction is assumed to follow the pathway:

$* + H_2O \rightarrow *OH + H^+ + e^-$

$*OH \rightarrow *O + H^+ + e^-$

$*O + H_2O \rightarrow *OOH + H^+ + e^-$

$*OOH \rightarrow O_2 + * + H^+ + e^-$

However, on CoOOH we find that the proton on the *OOH intermediate can sometimes be transferred spontaneously to a neighbouring *OH.

The adsorption energies of the reaction intermediates calculated by DFT ($E_{ads,DFT}$) are corrected for changes in zero point energy (ΔZPE) and entropy (– TΔS) and the effect of an applied potential (U) is calculated using the computational hydrogen electrode,[10] i.e. the free energy of adsorption is calculated as:

$$\Delta G_{ads}(U) = E_{ads,DFT} + \Delta ZPE - T\Delta S - neU$$

Where n is the number of electrons involved in the reaction.

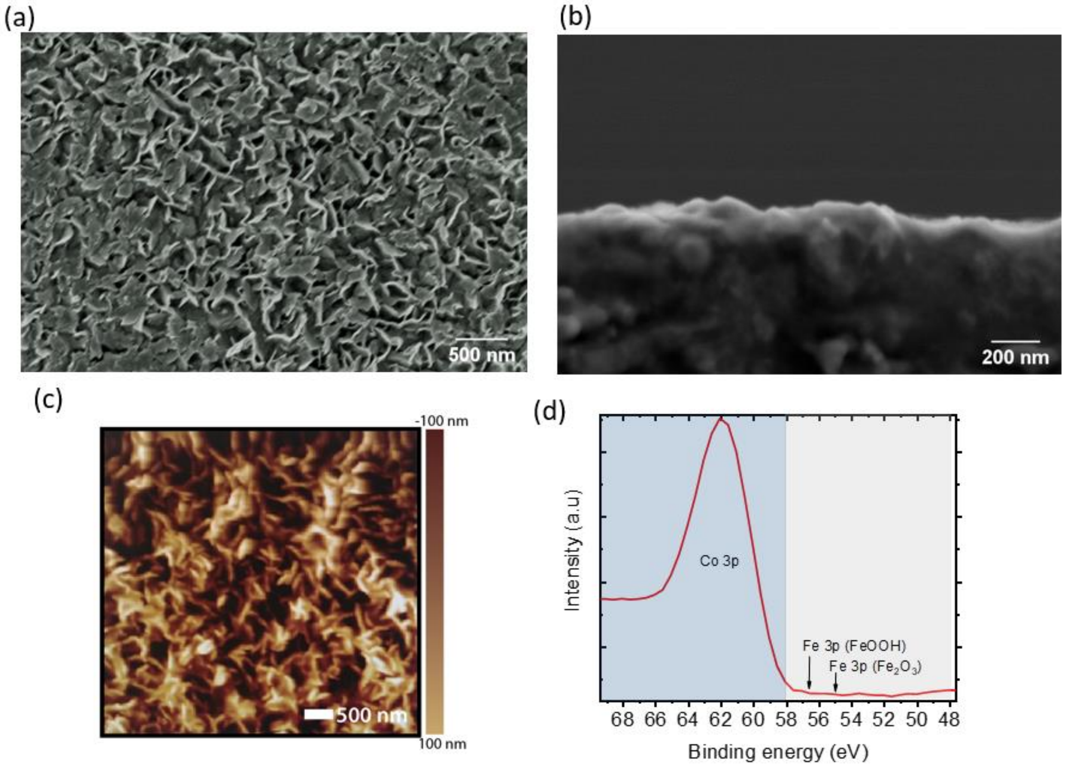


*Figure S1.* Microscopy of CoOOH films. (a) Top-down SEM. (b) Cross sectional SEM false coloured to highlight the CoOOH layer in grey. (c) AFM. (d) Co and Fe 3p XPS spectra. This region is chosen as the Fe 3s and 2p regions overlaps with peaks from Sn emission from the FTO substrate.

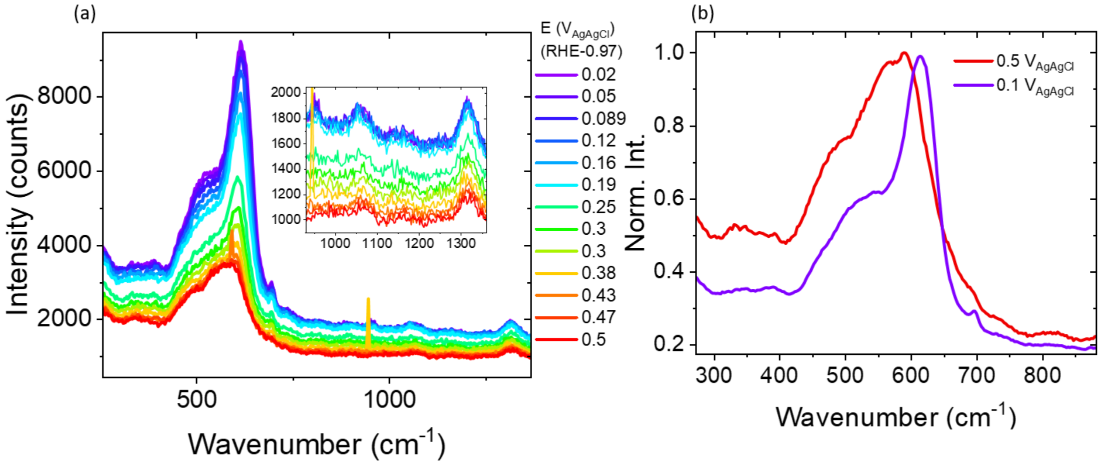


Figure S2. (a) In situ-surface enhanced Raman spectra of CoOOH at a series of applied potentials showing the Co-O breathing mode. The change in signal amplitude is a result of the differential absorption changes in the film at increasing applied potential (see SEC data in main paper). Inset: 1100-1300 wavenumbers region previously suggested by Moysiadou to show a bridging superoxo intermediate [5] shows no discernible broad signal between 1100-1300 wavenumbers growing with applied potential. (b) Normalised spectra showing the change in breathing modes consistent with the conclusions of Bell and Co-workers, indicating that a comparable structure is produced herein. [4] Conditions: purified 0.1 M KOH, Pt wire counter electrode sat.d KCl Ag/AgCl reference electrode.

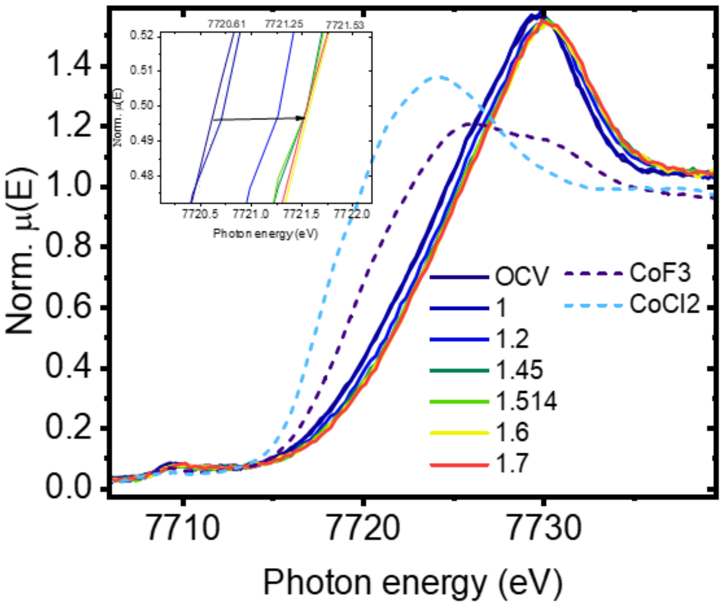


Figure S3 In-situ Co K-edge XAS spectra of CoOOH at various applied potentials with inset showing a slight shift. Note the sample was cycled before study. Two standards are shown to guide the eye to the characteristic high energy absorption edge of cobalt oxyhydroxides[6]

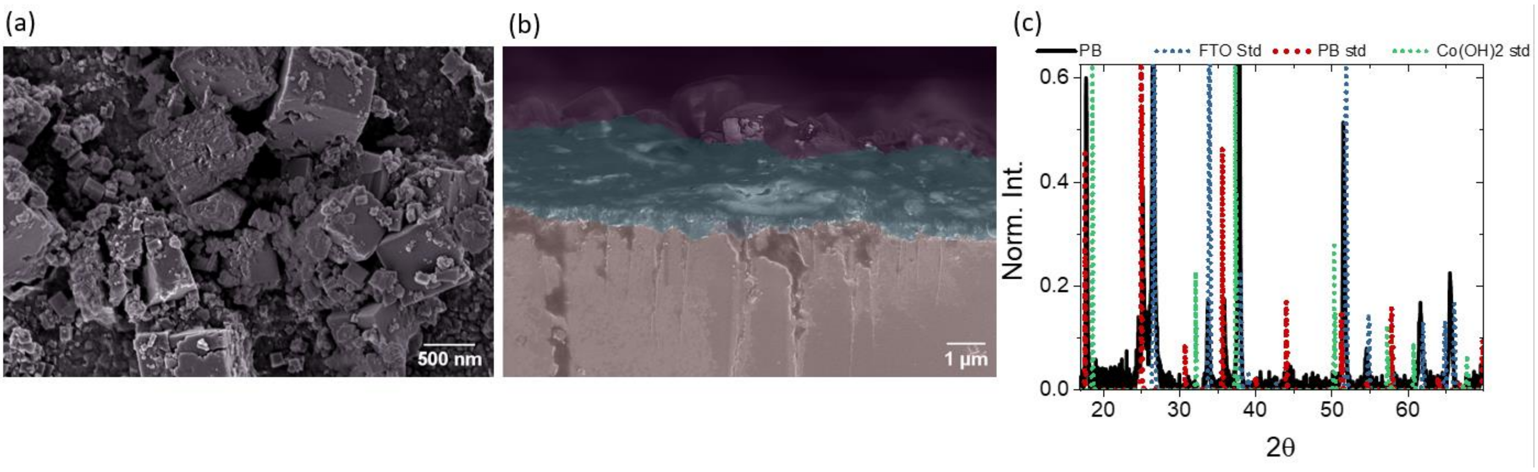


*Figure S4. (a)* Top Down SEM image of CoFe-PB. (b) Cross sectional SEM image false coloured to highlight the CoFe-PB in purple the background. (c) XRD pattern of CoFe-PB films shown alongside standards for FTO, CoFe-PB and $Co(OH)_2$

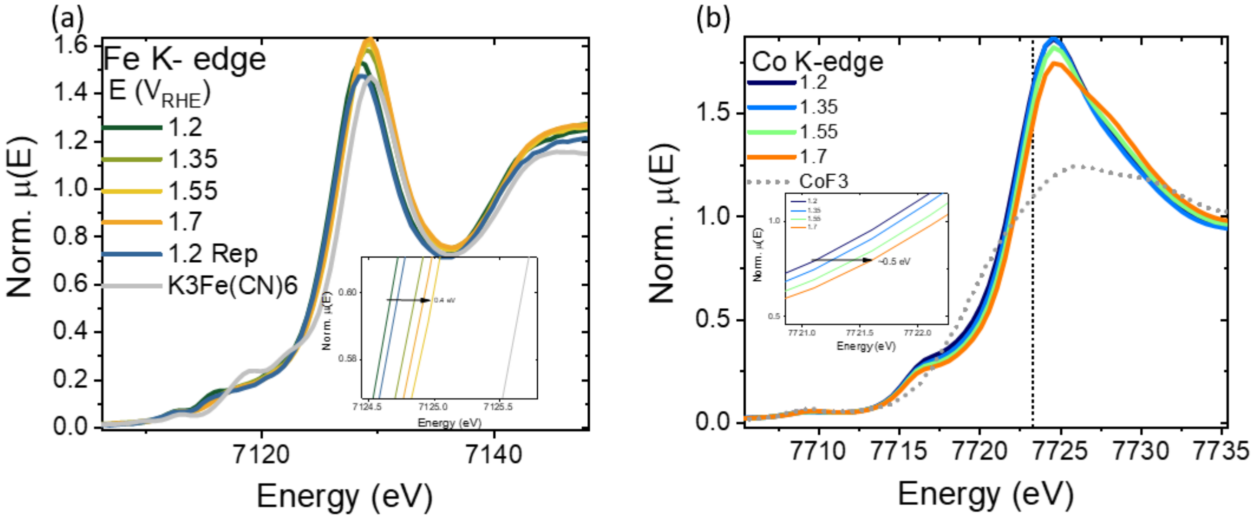


*Figure S5.* Fe (a) and Co (b) K-edge spectra of CoFe-PB at a series of applied potentials (vs RHE)

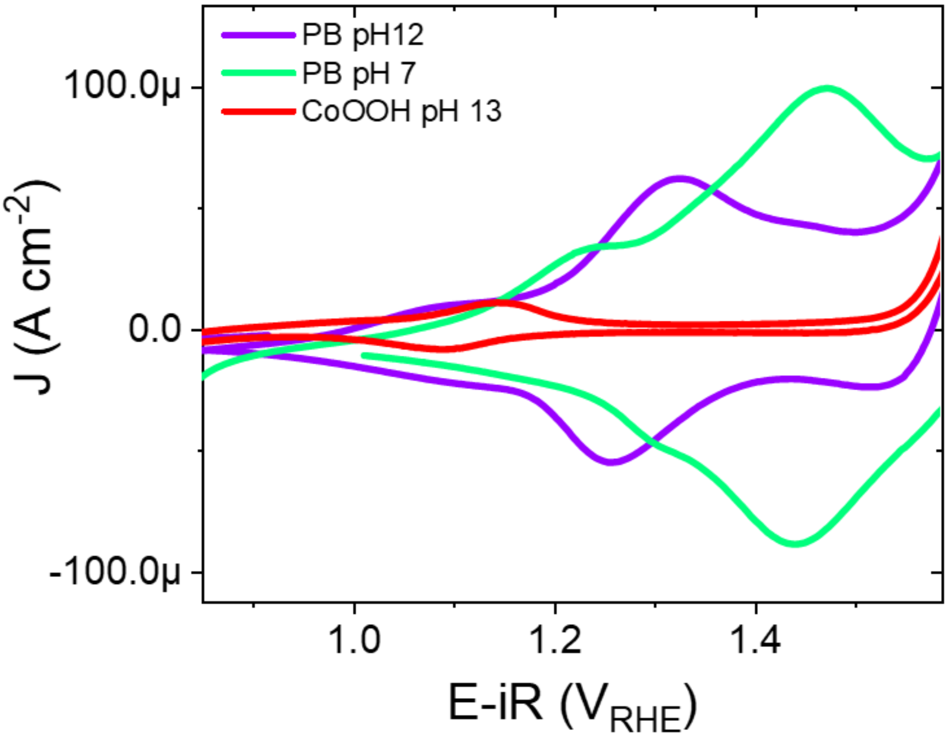


*Figure S6. CVs of CoFe-PB at variable pH. in comparison to CoOOH (0.1 M KOH).* Note CoFe-PB shows immediate and irreversible changes at pH 13 thus pH 12 is used as a comparison.

**S7 Calculation of component spectra from SEC data and an explanation of the physical significance of fitted data and calculation of redox currents.**

The absorbance for a given species (*i*) is

$$A_i = -\log\frac{I}{I_0} = \epsilon_i(\lambda)c_i l$$

Where $I_0$ is incident intensity of light, I is the transmitted intensity of light, $\epsilon_i$ is the molar absorptivity of species *i,* in units of $Mol^{-1}\ dm^3\ cm^{-1}$, $c_i$ is the concentration in units of $Mol^1\ dm^{-3}$ and $l$ is the path length in units of *cm.*

**Note on the units used for SEC.**

However, in spectroelectrochemistry, the dimensions of the $\epsilon$ do not lend themselves to the experiment, as transformations of solids are measured, rather than solutions with a 1 cm path length. Here, the absorbance of a solid film in 1 $cm^2$ area (not concentration) is measured and correlated with charge. To address this problem, we modify the units of concentration and extinction by (1) converting units relating to volume from $dm^3$ to $cm^3$ (i.e. division and multiplication by 1000 to convert C and $\epsilon$). (2) multiplication of the units of $l$ into $c$ to give an area density in units of Mol $cm^{-2}$. (3) Conversion of molar units to an equivalent charge (assuming that one moiety is generated by a 1 $e^-$ couple) by multiplication and division by faradays constant. This re-expresses concentrations in terms of a charge density $Q_i$ (in units of $C\ cm^{-2}$ ) and extinction coefficients as coulometric attenuation coefficient in units of $cm^2\ C^{-1}$. We note that because the redox transitions in question are thought to be coupled to the generation of water, charge here does not imply an electrostatic charge on the electrode surface but rather, only monitors the number of electrons that have passed around the external circuit up until a potential U.

$$A_i = \varepsilon_i c_i l = \alpha_i Q_i$$

For a given conversion process:

$$nX \rightarrow (n-m)X + mY$$

The change in absorbance in terms of traditional concentrations and extinction coefficients is:

$$\Delta A = \epsilon_X(C_{Tot} - C_Y) + \epsilon_Y C_Y - \epsilon_X C_{Tot}$$

$$\Delta A = \epsilon_X(C_{Tot} - C_Y) + \epsilon_Y C_Y - \epsilon_X C_{Tot}$$

$$\Delta A = C_Y(\epsilon_Y - \epsilon_X) = C_Y \Delta\epsilon$$

Where $\epsilon_X$ and $\epsilon_Y$ are the extinction coefficients of species X and Y and $C_T$ is the total concentration of species present in the system, which is used to substitute the concentrations of X ($C_X$)in terms of the concentration of Y ($C_Y$) and the total concentration, with is a conserved quantity ($C_{Tot}$):

$$C_{Tot} = C_X + C_Y$$

Analogous equations can be written in terms of $\alpha_i Q_i$:

$$\Delta A = Q_Y(\alpha_Y - \alpha_X) = Q_Y \Delta\alpha$$

Where $\Delta\alpha$ is the coulometric differential attenuation coefficient, assuming a single electron couple. The conserved quantity here is not the total concentration but concentration converted to a charge assuming a single electron couple:

$$(when\ all\ X \rightarrow Y)\ Q_Y = Q_{Tot}$$

**Rationale for fitting using the example of a single redox process.**

The above equations state that differential spectra *do not a-priori give absolute* populations, but *rather track the number of interconverting moles (i.e. the charge passed).* ***Thus, differential absorption tracks the extent of a redox transition*** $(\boldsymbol{\theta})$***, not the absolute population.*** Even in this simple case, one would not know the value of $Q_T$ until the redox transition in complete (*when* $Q_Y = Q_{Tot}$*).* This can clearly be seen by defining $\theta$ for this simple process. Hereafter referring to $Q_Y$ simply as $Q$:

$$\theta = \frac{Q}{Q_{Tot}} = \frac{C_Y}{C_{Tot}}$$

Thus

$$Q_{Tot}\theta = Q$$

We can therefore write $\Delta A$ in terms of the extent of reaction:

$$\boldsymbol{\Delta A = Q\Delta\alpha = Q_{Tot}\theta\Delta\alpha}$$

The differential attenuation coefficient normalised by its peak value (hereafter $\overline{\Delta\alpha} = \frac{\Delta\alpha(\lambda)}{\Delta\alpha(\lambda_{peak})}$) can be extracted from the data. To show this, we re-write $\Delta A$ in terms of $\overline{\Delta\alpha}$:

$$\boldsymbol{\Delta A = Q_{Tot}\theta\overline{\Delta\alpha}(\lambda)\Delta\alpha(\lambda_{peak})}$$

If we now normalise $\Delta A$ by its peak value we may extract $\overline{\Delta\alpha}$:

$$\overline{\Delta A} = \frac{\Delta A(\lambda)}{\Delta A(\lambda_{peak})} = \frac{Q_{Tot}\theta\Delta\alpha(\lambda)}{Q_{Tot}\theta\Delta\alpha(\lambda_{peak})} = \overline{\Delta\alpha}$$

This shows that the normalised differential absorbance corresponds to the normalised differential coulometric actuation coefficient in the case of a single conversion process. Using this information we may devise a fitting procedure, as we can obtain. By grouping terms:

$$\beta = Q_{Tot}\theta\Delta\alpha(\lambda_{peak})$$

We see that:

$$\Delta A = \beta\overline{\Delta\alpha}$$

This is the rational for the fitting procedure used herein where we use $\overline{\Delta\alpha}$ to fit the data.

Using an experimentally determined normalised differential coulometric actuation coefficient (see following sections), one may fit the measured $\Delta A$ as a function of the applied potential (U). If, from a separate experiment, one may measure $\Delta\alpha(\lambda_{peak})$, then $Q$ can be extracted.

$$\frac{\beta}{\Delta\alpha(\lambda_{peak})} = Q_{Tot}\theta(U) = Q(U)$$

If one is able to see the process reach completion ($\theta = 1$), then one is also able to estimate $Q_{Tot}$ and thus $\theta$ at other potentials:

$$\frac{\beta}{\Delta\alpha(\lambda_{peak})\, Q_{Tot}} = \theta(U)$$

***<u>Note on the isolation of normalised differential coulometric actuation coefficient spectra (i.e. component spectra used for fitting) in the case of multiple redox processes.</u>***

In the case of the materials studies herein, multiple overlapping redox transitions are observed. The differential absorbance observed in a system exhibiting multiple processes is:

$$\Delta A = \sum_{\substack{Redox \\ Transitions \\ T_i}} Q_{T_i}(\mathrm{U})\Delta\alpha_{T_i}(\lambda)$$

Where $Q_{T_i}$ and $\Delta\alpha_{T_i}$ refer to the partial charge transferred and the differential coulometric actuation coefficient arising from the $i^{th}$ redox transition. The partial charges that are extracted from a given redox transition dominate different regions. If there exists a region where the charge extracted

arises from only redox process, the difference of two $\Delta A$ values evaluated in this region will be:

$$(\Delta\mathrm{A})_\delta = \Delta A(U) - \Delta A(U-\delta) = \sum_{\substack{Redox \\ Transitions \\ T_i}} Q_{T_i}(\mathrm{U})\Delta\alpha_{T_i} - Q_{T_i}(\mathrm{U}-\delta)\Delta\alpha_{T_i}$$

If in the region between U and $U - \delta\ if\ all\ transitions\ T_j \neq T_i)$ are not progressing (i.e. charge is only being extracted from one redox process), then all terms corresponding to other processes will be cancel:

$$(\Delta\mathrm{A})_\delta = Q_{T_i}(\mathrm{U})\Delta\alpha_{T_i} - Q_{T_i}(\mathrm{U}-\delta)\Delta\alpha_{T_i} = \Delta\alpha_{T_i}(\delta Q_{T_i})$$

Here we again have a linear correspondence between differential absorbance and charge via a single differential coefficient. This situation is analogous to the single conversion process shown above and it is trivial to show that in region where only one redox transition is proceeding that:

$$\overline{(\boldsymbol{\Delta A})}_{\boldsymbol{\delta}} = \frac{(\Delta\mathrm{A})_\delta}{(\Delta\mathrm{A}(\lambda_{\mathrm{peak}})_\delta} = \frac{\Delta\alpha_{T_i}(\lambda)}{\Delta\alpha_{T_i}(\lambda_{peak})} = \overline{\boldsymbol{\Delta\alpha}_{T_i}}$$

This form enables the extraction of the normalised differential coulometric actuation coefficient spectra. From this, the fitting procedure for multiple redox transitions can be obtained:

$$\Delta A = \sum_{\substack{Redox \\ Transitions \\ T_i}} = \beta_{T_i}\overline{\Delta\alpha_{T_i}}$$

In experimental data $(\Delta A)_\delta$ is approximated by subtracting $\Delta A$(U) and $\Delta A$(U-20mV) (hereafter $\Delta A_{\delta=20\ mV}$ or more simply $\Delta A_{20\ mV}$). A plot of $\Delta A_{20\ mV}$ is given for CoOOH and CoFe-PB in S7 (a) and (b) respectively below. In 7a, redox transition T1 is left out for clarity and a single characteristic dotted line is added characteristic of redox transition T1. We note that the transition from the characteristic spectrum of redox transition T2 (purple) to that of T3 (red) occurs at extremely positive potentials relative to the OER potential. The transition completes by ca. 1.35 $V_{RHE}$, after which a single normalised spectrum (T3) is observed. This indicates that at potentials above 1.35 $V_{RHE}$ incrementing the bias produces only conversion process; T3. 7b shows the differential spectra for CoFe-PB. Here, an initial broad spectrum (T1, purple) changes to an asymmetric spectrum previously observed in spectroelectrochemical experiments and attributed to precatalystic states.[11] This spectrum then broadens at potentials around the onset of OER to produce a third spectrum (redox 3), again consistent with previous findings. [11]

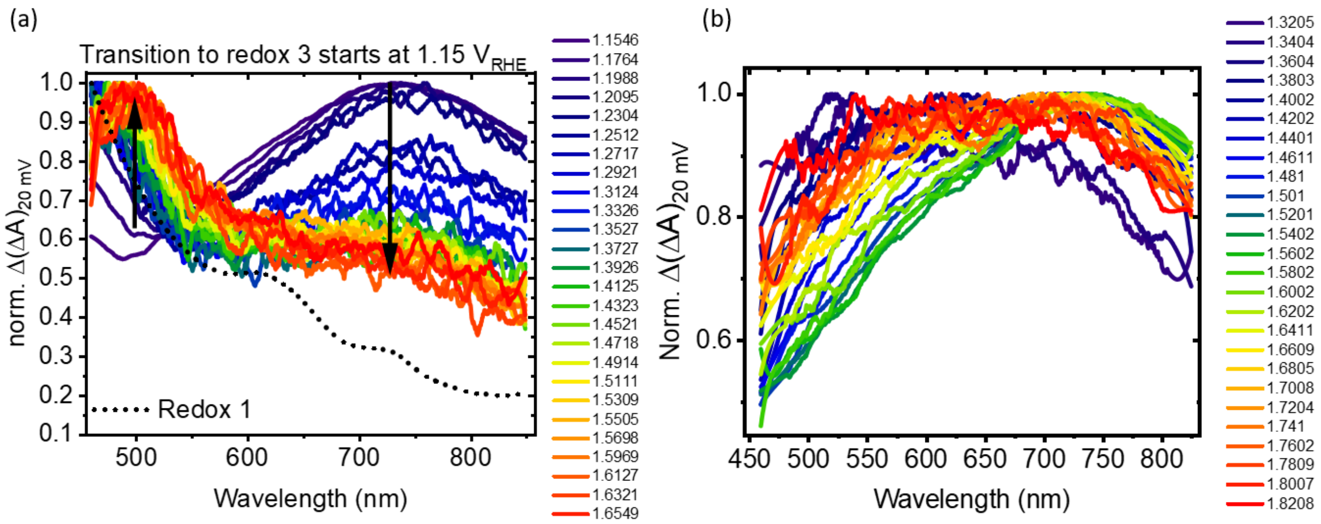


*Figure S7.* $\Delta A_v - \Delta A_{v-20mV}$ for CoOOH (a) and CoFe-PB (b). The spectra converge in two regions in both cases. For clarity spectra associated with the less significant component (redox transition 1) are not shown. Note the regions where the spectra converge.

**Note on the constraints used in Fitting**

From these spectra a sequential linear fitting procedure was applied with three key constraints: firstly, a spectrum cannot appear long before its component spectrum appears in Figure S7. This reflects the simple electrochemical reasoning that at an applied potential $U \ll U^0$ where $U^0$ is the standard redox potential a process, no interconversion is expected. A second constraint is that component spectra cannot decay as no loss of absorption is observed that would indicate the condition where $\Delta A$ is negative (i.e. the case when $\Delta\alpha = \alpha_2 - \alpha_1$, $\alpha_2 < \alpha_1$). Finally, there is some degree of spectral similarity of redox 1 and 3 in CoFe-PB. For redox 1, both the population change and signal become saturated and stop changing before the onset of redox 3. To aid in fitting redox 1 absorbance was constrained in this region and changes are purely associated with redox 3 (as for redox 1 $U \gg U^0$ applies, thus no change in absorbance should be observed). This last assumption is not necessary but aids in reducing noise during fitting and so is recommended but should be used with care. Fitting was performed using an in-house code written in Julia using gradient descent and the NLOPT package.

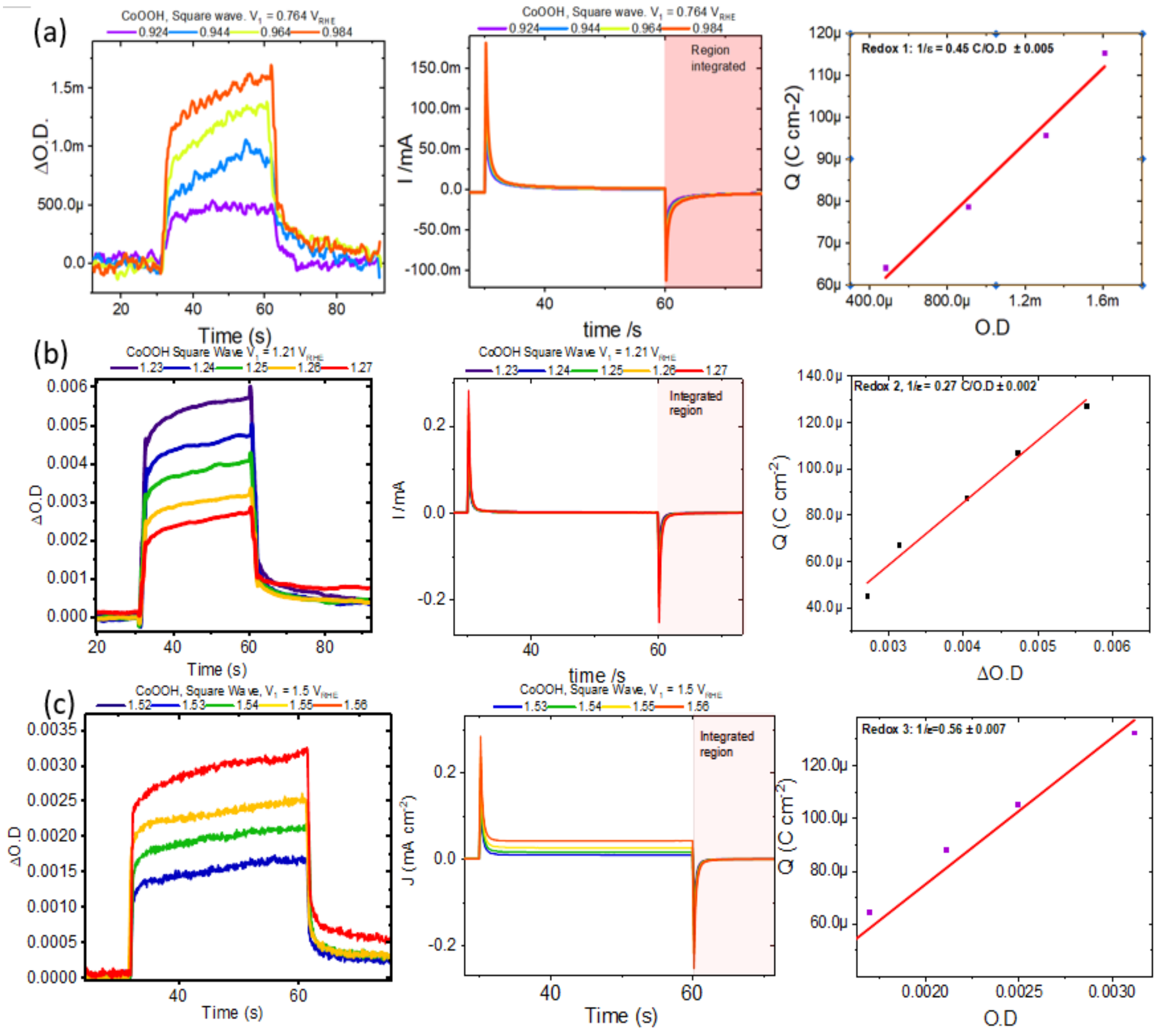


*Figure S8 Calculation of attenuation coefficients for redox transition T1 (a) ,T 2 (b) and T3 (c) for CoOOH.* **Left:** the differential absorbance generated by a square wave voltage, **Middle**: the resulting current transients, **Right** The correlation of charge with optical absorbance.

## S8 calculation of attenuation coefficients

The information above enables the determination of potential regions where only one redox transition contributes to the current such that:

$$(\Delta \mathrm{A})_{\delta} = \Delta\alpha_{T_i}(\delta Q_{T_i})$$

Holds true. In a region of applied potential where only one redox process takes place the differential attenuation coefficient can be calculated as the gradient of a straight line linking the change in concentration to the change in absorption.

$$\Delta A_{T_i} = Q_{Ti}\Delta\alpha_{Ti}(\lambda_{peak})$$

To calculate $Q$ we use a square wave voltametric technique in a region known to contain only one redox process (see top panel of Figure 3 in main text for justification of potentials as these clearly show small regions were only one process contributes to charge). Spectra were checked to confirm their similarity to the normalised differential coulometric actuation coefficient spectra. $\Delta\alpha_{Ti}(\lambda_{peak})$ is the slope of the peak differential absorption plotted against the charge obtained from integrating

the back transient of the current in the square wave voltammogram a single electron transfer results in interchange between species. This is shown for CoOOH in Figure S8 a-c below and CoFe-PB in Figure S8 d-e. As converting from $\Delta A_{T_i}$ to $Q_{Ti}$ requires division by $\Delta\alpha_{Ti}(\lambda_{peak})$ we present our results as $1/\Delta\alpha_{Ti}(\lambda_{peak})$ to facilitate easy conversion between differential absorbance and partial charge.

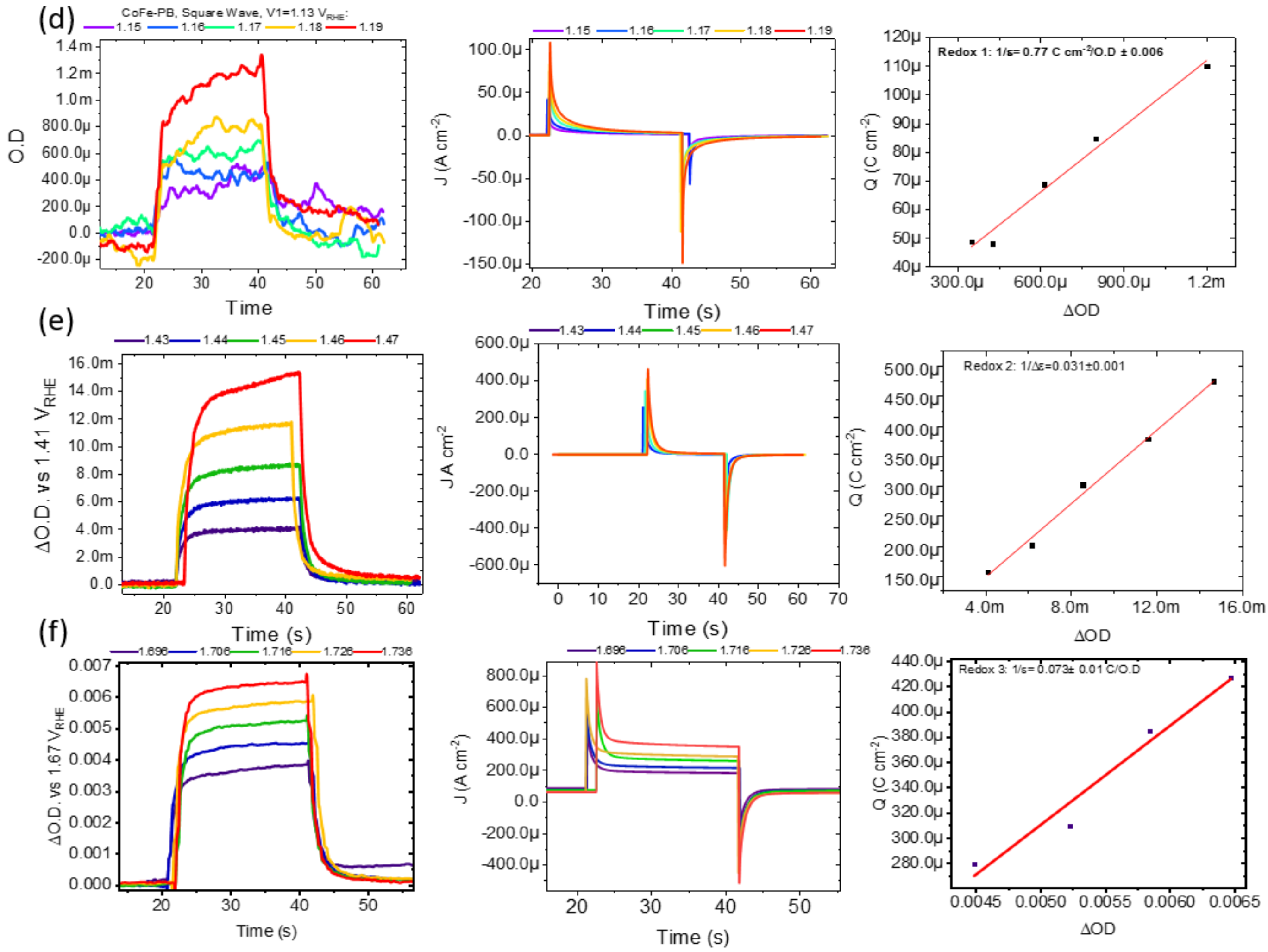


*Figure S8. Calculation of attenuation coefficients for redox transition T1 (d) , T2 (e) and T3 (f) for CoFe-PB.* **Left:** the differential absorbance generated by a square wave voltage, **Middle**: the resulting current transients, **Right** The correlation of charge with optical absorbance.

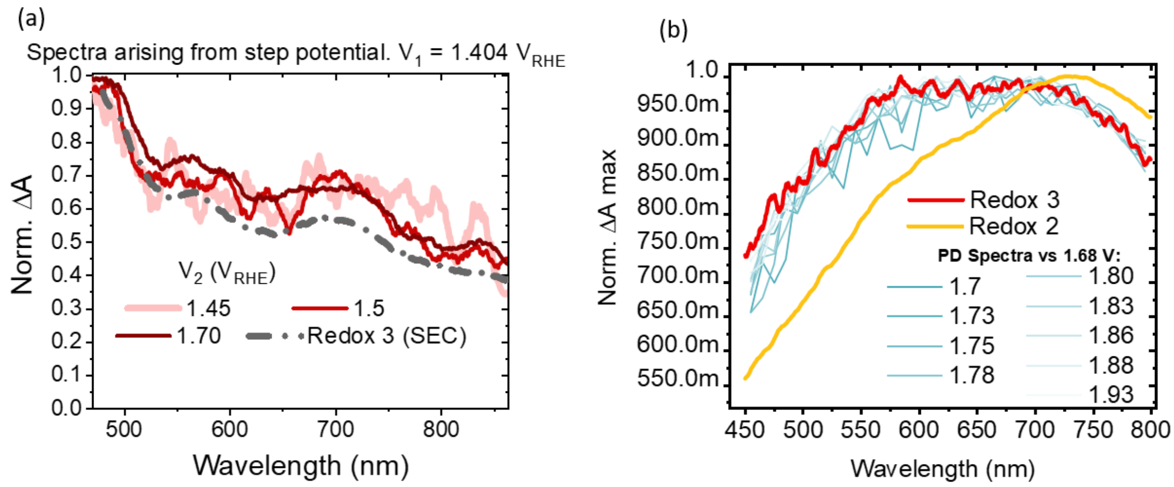


*Figure S9 Comparison of the differential absorbance spectra obtained at different step sizes to the normalised differential attenuation coefficient (component spectra) of redox transition T3 for CoOOH (a) and T2 and T3 CoFe-PB (b).*

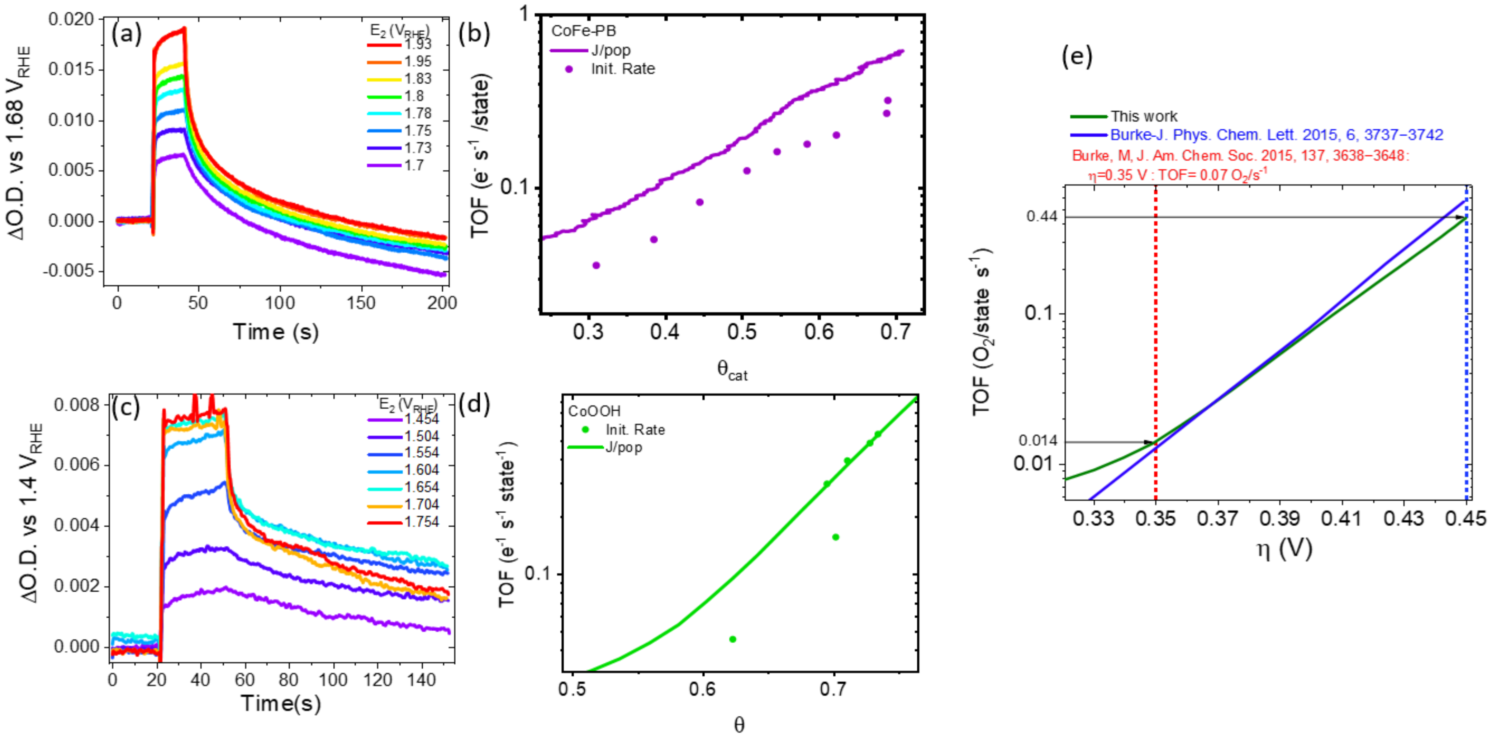


*Figure S10 (a) Step potential induced accumulation and open circuit decay kinetics of CoFe-PB at a series of increasing step potentials. (b) TOF as a function of coverage, comparing TOF calculated by diving current by partial charge and initial rate of optical decay (represented as a current* $J_{optical,intial\ rate}$ in the equations below) *by partial charge. (c) Step potential induced accumulation and open circuit decay kinetics of CoOOH at a series of increasing applied potentials, with corresponding of initial rate and current derived TOF comparison (d). (e) Comparison of TOF observed herein for CoOOH to those obtained by measurements by Burke et. al.*

**S8 Calculation of turnover frequency from initial rate**

As we measure partial charge of different surface redox processes, an apparent TOF can simply be obtained by dividing the OER current by the partial charge needed to generate a given coverage of the rate limiting intermediate ($\mathrm{Q_{T3}}$):

$$apparent\ \mathrm{TOF} = \frac{\mathrm{J_{OER\ measured\ by\ the\ potentiostat}}}{\mathrm{Q_{T3}}}$$

If the measured intermediate was inactive. One would still be able to measure this value, although the result would be meaningless. However, if the intermediate is active then the initial rate of decay of the intermediate under open circuit condition should be commensurate to the apparent TOF, as both are should measure inherent activity. As $\Delta\mathrm{A}$ is a measure of partial charge the initial rate of decay is proportional to a current:

$$\frac{\mathrm{d\Delta A_{T3}}}{\mathrm{dt}} \cdot \frac{1}{\Delta\alpha_{\mathrm{T3}}} = \frac{\mathrm{dQ_{T3}}}{\mathrm{dt}} = \mathrm{J_{optical,intial\ rate}}$$

With the TOF equal to

$$\mathrm{Inital\ rate\ TOF} = \frac{\mathrm{J_{optical,inital\ rate}}}{\mathrm{Q_{T3}}}$$

$\mathrm{Q_{T3}}$ is obtained by adding the partial charge of redox transition T3 at the starting potential (obtained from spectroelectrochemistry) to the jump in absorbance from applying the step in potential, converted to a partial charge. $\mathrm{J_{optical,inital\ rate}}$ is the initial rate of the optical signal, obtained by a similar linear fitting, converted into a current. Note, this is a state specific turnover frequency and is independent of the number of states involved in the RDS. If one assumes that one state is present in the RDS, as is indicated from our data then the $O_2$ turnover frequency is calculated by dividing the quoted numbers by 4 in order to compare directly to the literature.

**S11. Fitting of coverage and current to electroadsorption isotherms and BEP rate equations**

In S11a, the measured coverage as a function of potential is fitted using two electroadsorption isotherms for CoFe-PB (top panel) and CoOOH (bottom panel). The electrochemical analogue of Lanmguir adsorption (Lanmguir electroadsorption), and Frumkin electroadsorption. These equations differ by only one term, r/F*θ, which describes the change in adsorption enthalpy as coverage increases. Note that because of this term, the half wave potential no longer corresponds to the standard potential as at half coverage the Frumkin equation reduces to $E_{1/2}=E^0+0.5r/F$. In S11b, activation energy considered to be linear function of coverage as a result of the BEP theory. Here $E_A=a\theta+b$. The *"a"* parameter here is the BEP coverage coefficient it links the change in activation energy to the change in coverage. The "b" parameter is in principle the activation energy at zero coverage. However, in this case, "b" the fitted value of b is strongly affected by k, the calculated Eyring pre-factor. This value cannot be fitted by must rather be calculated according to version of the Eyring equation modified by Nhong et al.[12]

$$k = \frac{4|e|k_B T}{h} \mathrm{N}$$

Where $e$ is the elementary charge in C, $k_B T$ is the thermal energy at room temperature in eV, h is Plank's constant in eVs and N is the total number of available sites, which we calculate from our optical data to be 1.26x$10^{16}$ $cm^{-2}$ and 1.5x$10^{16}$ for CoOOH and CoFe-PB which corresponds to around 5 and 6 x $10^{10}$ C $s^{-1}$ $cm^{-2}$ respectively (to do this convert the completing charge of redox T2 into a number density using Faraday's and Avogadro's constants) . These numbers are fixed parameters during the fitting procedure, however any error in this calculation very strongly effects the fitted value for b and is thus we do not consider our values of b to be true values without an external measurement of activation energy to confirm this. This is the subject of an ongoing study.

Given the implicit potential dependence of J in this model i.e. $J(\theta(U))$. One may calculate the Tafel slopes empirically by simple substitution. $\theta(U)$ herein is given by adsorption isotherms whilst the $J(\theta)$ is empirically determined fit of current versus coverage, shown in Figure S11 b for CoFe-PB (top panel) and CoOOH (bottom panel).

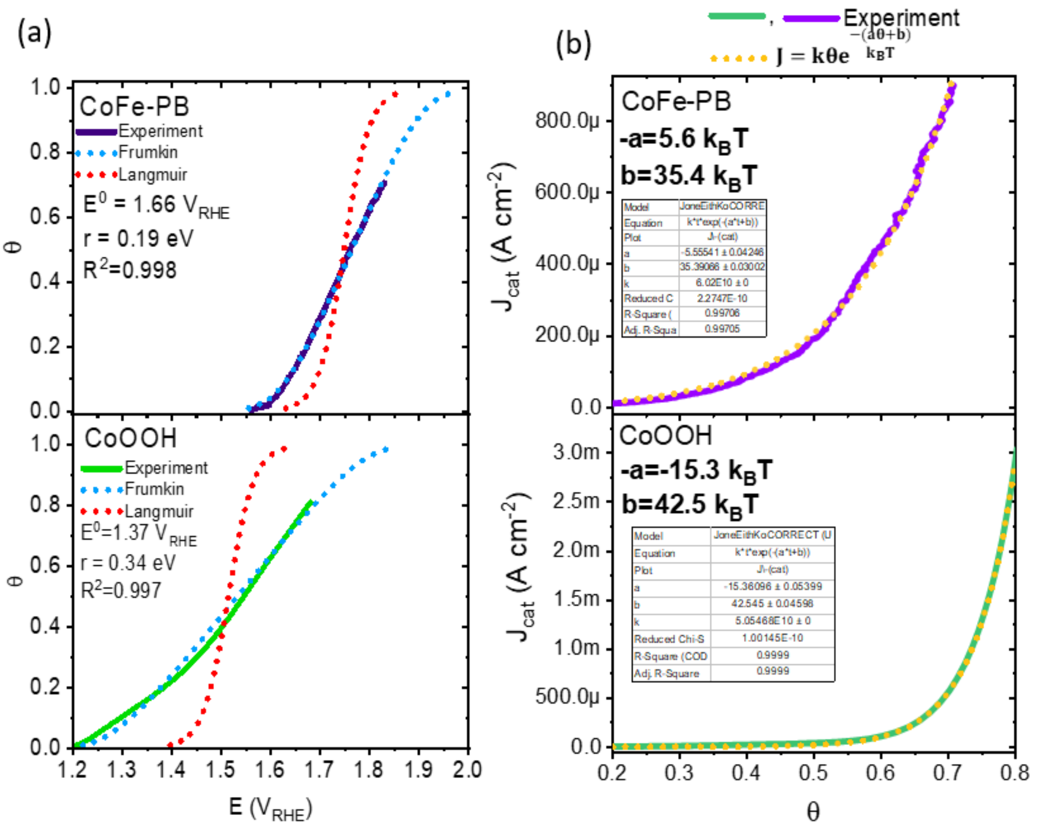


*Figure S11. (a) Fitting of potential as a function of coverage of CoOOH (bottom) and CoFe-PB (top) to frumkin and Langmuir electroadsorption isotherms. (b) Fitting OER current to a BEP driven rate equation for CoOOH (bottom) and CoFe-PB (top).*

## CoOOH Calculations

The lattice constants of bulk β-CoOOH were optimized to a=b=2.91Å, c=13.04Å. This crystal structure is the most stable phase at the potential of OER.[13,14] Three different surfaces were considered following the results of Bell and co workers[14]: the 0001 surface, parallel to the CoOOH sheets, and the $01\bar{1}2$ and $10\bar{1}4$ edge terminations. The surfaces are modeled by a slab with five layers of Co atoms, with the bottom 2 layers fixed in the position of the bulk atoms. A minimum of 20 Å of vacuum is added between periodic images of the slabs to avoid interactions. All calculations are relaxed until the forces on all atoms are below 0.05 eV/Å.

### S12: 0001 surface

The 0001 surface is modelled as a 2x2 surface, and the Brillouin zone is sampled by 4x4 k-points. The pourbaix diagram of the considered surface terminations is shown in Figure S12a.

In agreement with Bell and co workers[7] we find that the lowest energy surface at relevant potential for OER is fully deprotonated. Calculating the OER on this surface shows that the reaction is limited by the formation of *OOH which requires a potential of 2.66 V. The energy diagram is shown in Figure S12b, at a potential of 1.62V. This result is in qualitative agreement with the calculations in ref[14] although the limiting potential found in these calculations had a smaller value of 2.03 V. We conclude that the 0001 surface is not active for OER, which is also in agreement with experimental observations suggesting that OER happens primarily at edge sites.[15] For this reason, the interaction between adsorbates on the 0001 surface has not been studied further.

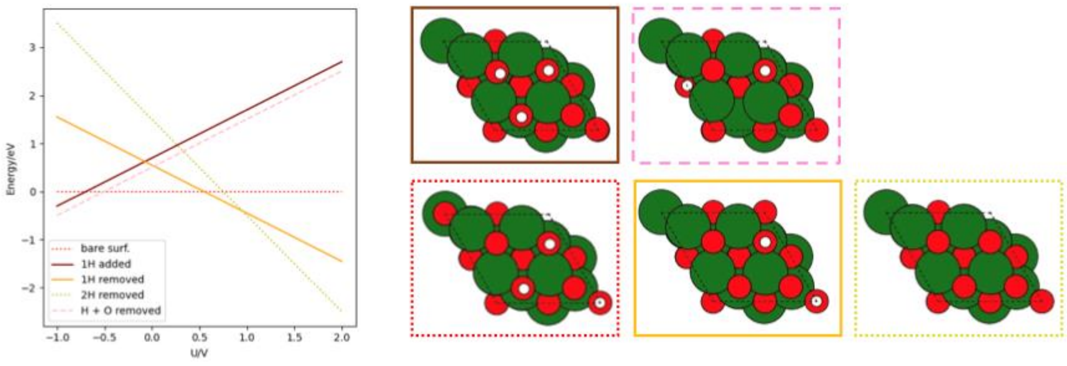


Figure S12a: Pourbaix diagram for the 0001 surface (left) showing the most stable surface as a function of the potential. The investigated surface structures (right) are drawn with the colour of the frames matching the colours of the lines in the pourbaix diagram. Co atoms are green, O is red and H is white.

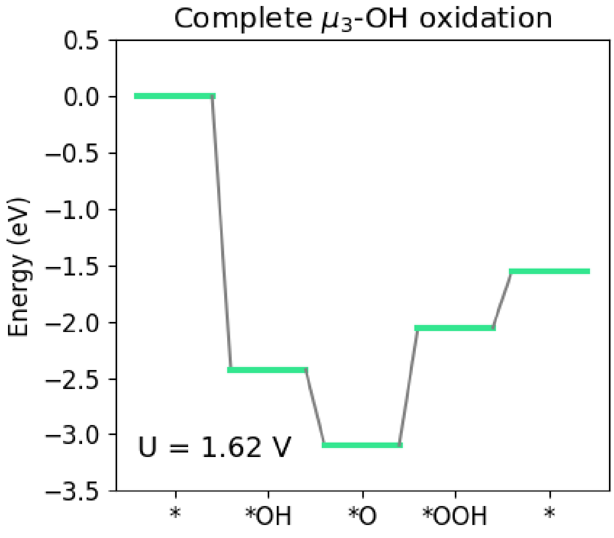


Figure S12b: Energy diagram for OER on the $\mu_3$ site of the 0001 surface at a potential of 1.62 V. The empty $\mu_3$ site is denoted by *.

## S13, the $01\bar{1}2$ surface

The $01\bar{1}2$ surface is modelled in a 3x1 unit cell, such that interactions between adsorbates along the edge of the CoOOH sheet can be probed. The brilluoin zone is sampled by 3x4 k-points. The surface termination at different potentials is probed by considering the full coverage adsorption of *OH, *O and *$H_2O$ on the coordinatively unsaturated site (top site, referred to as $\mu_1$ in the following) of the stoichiometric surface, as well as deprotonation of the OH groups residing in bridge sites on the side of the surface Co atoms (referred to as $\mu_2$-OH). The pourbaix diagram for the $01\bar{1}2$ surface is shown in Figure S13a, showing that OH is adsorbed on the $\mu_1$-sites of the stoichiometric surface at ca. 1.2 V, and the bridging $\mu_2$-OH groups of the resulting $\mu_1$-OH + $\mu_2$-OH surface are deprotonated around 1.5 V to form the $\mu_1$-OH + $\mu_2$-O surface.

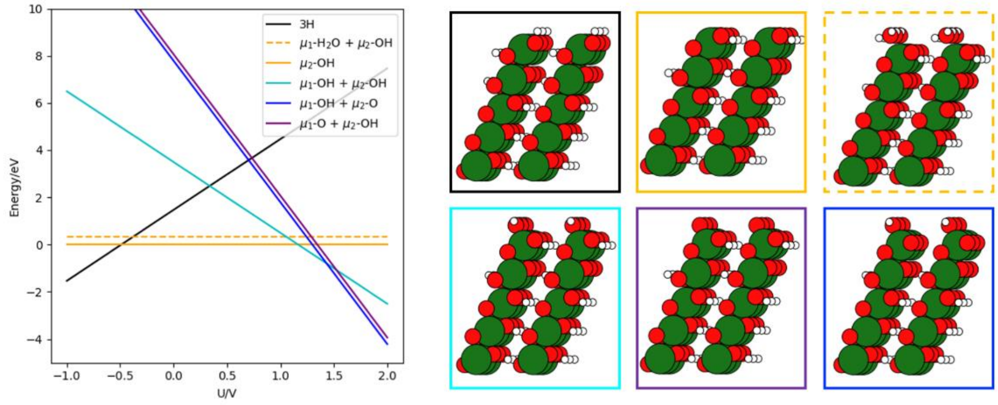


Figure S13a (left): Pourbaix diagram for the $01\bar{1}2$ surface (left) showing the most stable surface as a function of the potential. The investigated surface structures (right) are drawn with the colour of the frames matching the colours of the lines in the pourbaix diagram. Co atoms are green, O is red and H is white. (right) surface structures, color coded to match the colors in the left panel.

To investigate the interaction between neighbouring sites we calculate the energy of the individual sites of a supercell, which are initially equivalent, in the two relevant transitions ($\mu_1$-* --> $\mu_1$-OH and $\mu_2$-OH --> $\mu_2$-O). As seen in Table S1, the energies of oxidation each site in the reaction differ, due to the interaction between the adsorbates. We calculate the free energy of the reaction at half coverage ($\Delta G(\theta=1/2)$) as the average energy of the smallest and the largest reaction step, for comparison with the experimentally determined $E_0$. The width, *r*, also given in Table S1, is calculated as the difference in energy between these two steps and corresponds to the interaction parameter in the Frumkin isotherm. The computed values for the first transition (($\Delta G(\theta=1/2)$=1.19 eV, *r*=0.13 eV) match very well with the experimentally measured values for redox 2 (E0 = 1.17, r=0), while the values for the second transition ($\Delta G(\theta=1/2)$=1.44 eV, *r*=0.37 eV) match the values of the measured redox 3 ($\Delta G(\theta=½)$ = 1.5, r=0.34 eV). These results are summarized in Figure 7 of the main paper.

*Table S1: Energies for the stepwise transitions between stable surface structures on the* $01\bar{1}2$ *surface, and corresponding parameters for the Frumkin isotherm.*

| | 1st | 2nd | 3rd | $\Delta G(\theta=1/2)$ (eV) | *r (eV)* |
|---|---|---|---|---|---|
| * → $\mu_1$-OH | 1.14 | 1.12 | 1.25 | 1.19 | 0.13 |
| $\mu_2$-OH → $\mu_2$ -O | 1.26 | 1.42 | 1.62 | 1.44 | 0.37 |

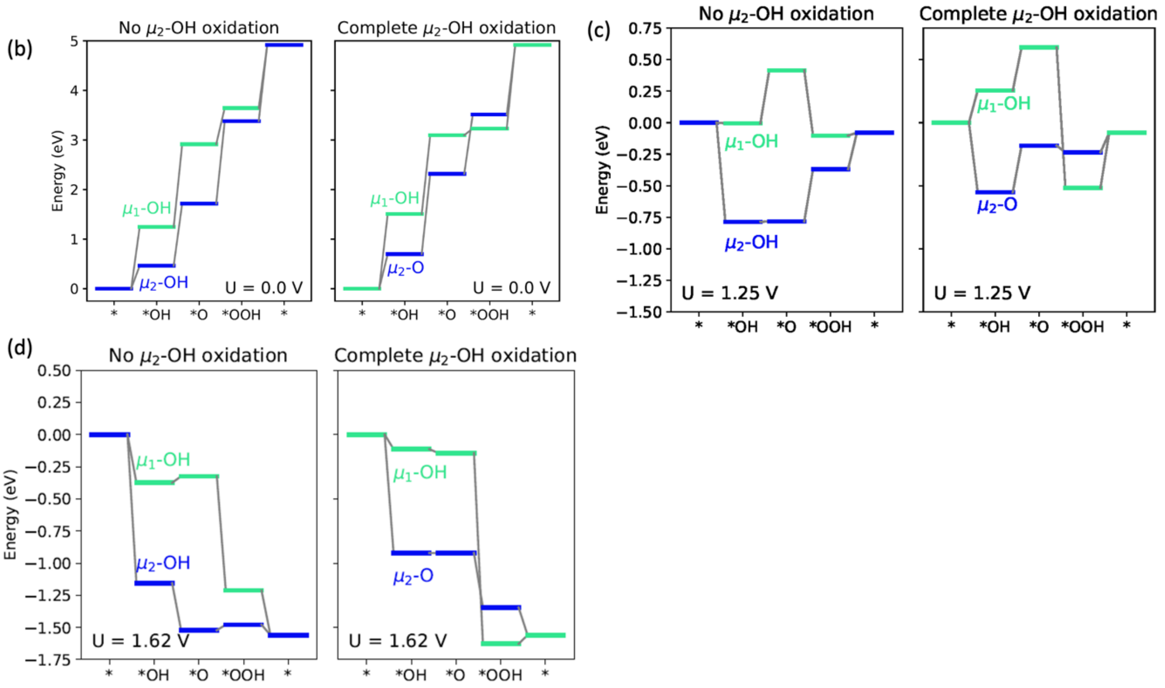


*S13-b-d.* Comparison of energy of the intermediates of on $\mathbf{01\bar{1}2}$ OER when $\mu_2$ sites are covered with OH (left) and by O (right at (b) 0 $V_{RHE}$ and (c) 1.25 $V_{RHE}$ (d) 1.62 $V_{RHE}$

**OER intermediates at various potentials on the $01\bar{1}2$ surface**

The strong interaction observed for the last transition is expected to have significant influence on OER. The intermediates of OER at a series of applied potentials with and without $\mu_2$-OH oxidation is shown above in Figure S13b-d. At 0 V (S13b), both surfaces are technically unstable and the reaction is uphill due to the absence of strong driving potential. However, modulation of the *O intermediate with respect to *OH can clearly be seen when $\mu_2$ is covered by *O instead of *OH. This destabilization also affects the $\mu_1$ site (shown in cyan). At a driving potential of 1.25 V, close to the thermodynamic

potential, the $\mu_2$-*OH covered surface is stable. The resulting energy diagram shown in Figure S13c demonstrates a significant overpotential for both sites. The modulation of intermediate energies by exchanging $\mu_2$-*OH for $\mu_2$-*O at the same potential is perhaps most clearly seen at this potential. Once all the $\mu_2$-OH is converted to $\mu_2$-O at a potential of 1.62 V the barrier for OER is minimal (Figure S13d). We note that these results are different to the ones obtained in ref[14], where a significant barrier for OER was also found on this surface. The reason for this may be that a different surface structure was considered, and deprotonation of the $\mu_2$-OH sites was not investigated.

**Calculation of the theoretical Frumkin isotherm.**

Given the estimated values of $\Delta G(\theta=1/2)$ and r, a theoretical Frumkin electroadsorption isotherm for these processes can be plotted from our DFT analysis. Here, the enthalpy of adsorption is considered to be a Langmuir electroadorption model modified by a coverage dependent interaction term, $r\theta$, (i.e. $U = E^0 + \frac{r}{F}\ln\left(\frac{\theta}{1-\theta}\right) + \frac{r}{F}\theta$), leading to an interaction dependent standard potential at $E^0 = E_{1/2} - \frac{r}{2F}$.[16]

**S14: $10\overline{1}4$ surface**

The $10\overline{1}4$ surface is modelled in a 1x3 unit cell, such that interactions between adsorbates along the edge of the CoOOH sheet can likewise be investigated for this surface. The brilluoin zone is sampled by 4x3 k-points. The surface termination at different potentials is probed by considering the full coverage adsorption of *OH, *O and *$H_2O$ on the stoichometric surface, as well as deprotonation of OH sites at the surface. On the $10\overline{1}4$ surface the adsorbates on the side of the sheets are coordinating to a single Co atom ($\mu_1$-OH, top-style adsorption) while the adsorbates on the top of the surface adsorb in a bridge configuration between two Co atoms ($\mu_2$-OH). The pourbaix diagram for the $10\overline{1}4$ surface is shown in Figure S14a, showing that the most stable stoichometric surface is covered by $\mu_2$-OH and empty $\mu_1$ sites, but adsorption of water on this surface to form $\mu_2$-OH + $\mu_1$-$H_2O$ results in a small stabilisation. The water is converted to $\mu_1$-OH at ca. 0.9 V and the $\mu_2$-OH groups of the surface are deprotonated around 1.5 V.

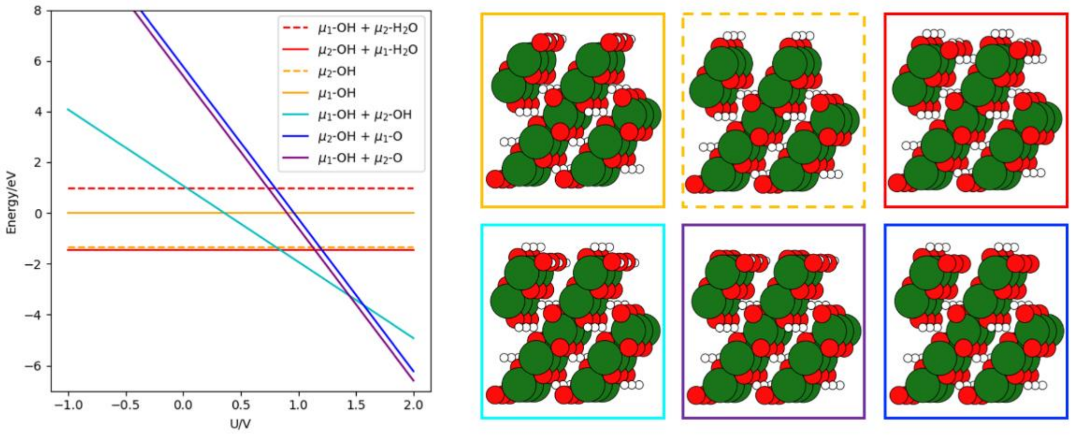


Figure S14a: (left) Pourbaix diagram of the $10\overline{1}4$ surface (left) showing the most stable surface as a function of the potential. The investigated surface structures (right) are drawn with the colour of the frames matching the colours of the lines in the diagram. Co atoms are green, O is red and H is white. (right) Illustrations of the surfaces corresponding to each line in the lefthand panel.

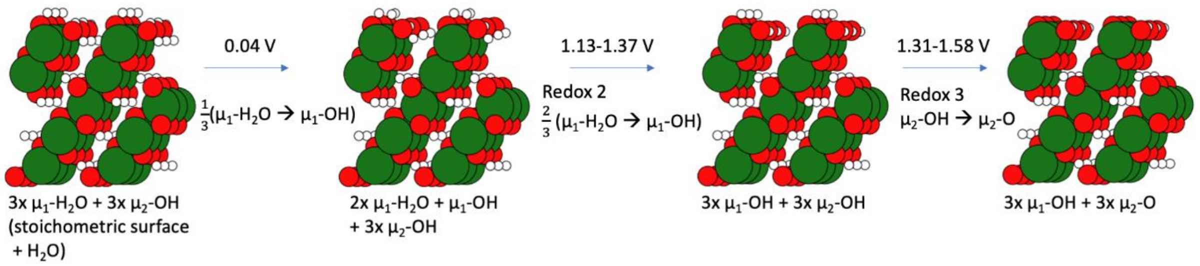


Figure S14b: Summary of the surface transitions of the $10\overline{1}4$ surface.

The interaction energy is again calculated by calculating the energy of the individual steps in the two relevant transitions ($\mu_1$-$H_2O$ --> $\mu_1$-OH and $\mu_2$-OH --> $\mu_2$-O). The results are shown in Table S2 together with the corresponding values of ΔG(θ=1/2) and *r* for the Frumkin isotherm. The results from the first transition suggest that 2$\mu_1$-$H_2O$ + $\mu_1$-OH + 3$\mu_2$-OH becomes the most stable structure at 0.04 V, and the remaining $\mu_1$-$H_2O$ are transformed to $\mu_1$-OH at much higher potentials (ΔG(θ=1/2) of 1.25 V), slightly higher than the first transition on the $01\overline{1}2$ surface. The second transition then starts immediately, and is completed at 1.57 V, with a ΔG(θ=1/2) similar to that of the second transition on the $01\overline{1}2$ surface. A considerable width is observed for both transitions. The transitions are summarized in Figure S14b.

*Table S2: Energies for the stepwise transitions between stable surface structures on the* $10\overline{1}4$ *surface and corresponding parameters for the Frumkin isotherm.*

| | 1st | 2nd | 3rd | ΔG(θ=1/2) | *r* |
|---|---|---|---|---|---|
| $\mu_1$-$H_2O$ →$\mu_1$-OH | 0.04 | 1.37 | 1.13 | 1.25[a] | 0.24[a] |
| $\mu_2$-OH → $\mu_2$-O | 1.31 | 1.58 | 1.45 | 1.44 | 0.27 |

[a] The values of ΔG(θ=1/2) and *r* are calculated from the 2nd and 3rd step only, since the first step is happening at much lower potential.

**OER Intermediates of the $10\bar{1}4$ surface**

We calculate the OER for the $\mu_1$-OH + $\mu_2$-OH and $\mu_1$-OH + $\mu_2$-O covered surfaces at both the $\mu_1$ and $\mu_2$ sites and plot the results in Figure S13c. The potentials of 1.25 V for the $\mu_1$-OH + $\mu_2$-OH surface and 1.62V for the $\mu_1$-OH + $\mu_2$-O surface are chosen to match the potentials of the energy diagrams for the $01\bar{1}2$ surface in Figure S12c-d, but since the transitions happen at similar potentials for the two edge-

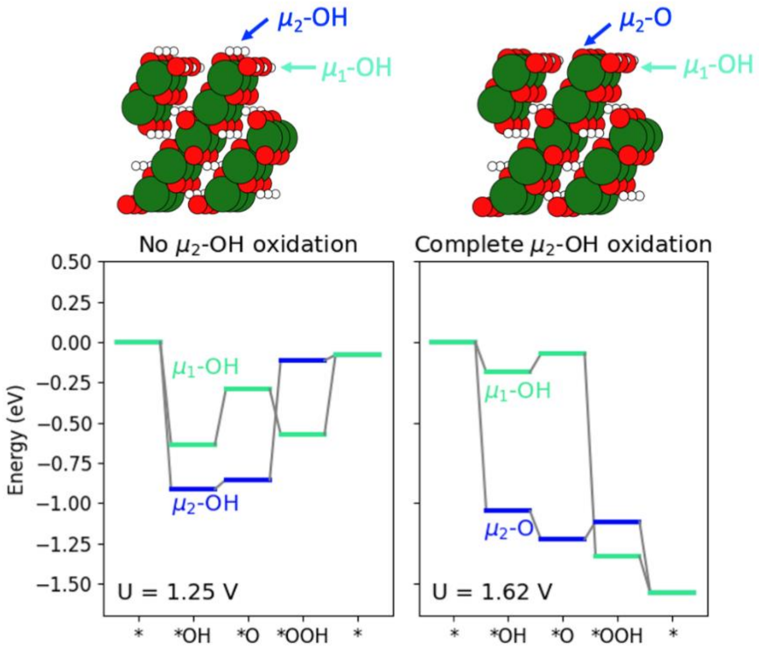


Figure S14c: Energy diagram for OER on the $\mu_1$-OH + $\mu_2$-OH CoOOH $10\bar{1}4$ surface (left), and the $\mu_1$-OH + $\mu_2$-O surface (right), considering the two different possible active sites. Sites indicated with coloured arrows in the structures correspond with the colours of the energy diagrams below. Note that for the reactions on the $\mu_1$-OH site, it is favourable for the OOH intermediate to transfer a proton to a neighbouring $\mu_1$-OH to form $\mu_1$-$O_2$+ $\mu_1$-$H_2O$, while reactions on the $\mu_2$-OH/$\mu_2$-O site go through OOH.

terminated surfaces these values are also representative of the potentials at which these $10\bar{1}4$ surfaces are stable. The energy diagram for the $\mu_1$-OH + $\mu_2$-OH surface demonstrates a significant overpotential for both sites, and the formation of *O will therefore begin before the reaction can proceed. $\mu_2$-OH is converted to $\mu_2$-O at a potential of 1.57 eV and the partly O-covered surface needs a potential of 1.73 eV for OER to happen on either of the two types of surface site. The calculated OER potential is similar to the one calculated for this surface in ref[14], although the preferred surface structure and reaction pathway found in this work is different.

The prevalence of the different surface facets in our CoOOH crystals is unknown, however the overall trends of the $01\bar{1}2$ and $10\bar{1}4$ surfaces show clear similarities and correspond well with the experimental observations. The surfaces get covered by OH on both $\mu_1$ and $\mu_2$ sites at a relatively low potential (1.11-1.37 V), matching well with the experimentally determined potential of redox 2. However, before OER becomes possible on this surface the $\mu_2$-OH sites (on top of the surface for the $10\bar{1}4$ surface and on the side for the $01\bar{1}2$ surface) are gradually deprotonated in a wide potential range, matching the wide redox peak of redox 3 and the accumulation of states before the OER onset. Once the deprotonation of these sites is completed at a potential of ca. 1.6V the resulting surface has a small barrier for OER, and the reaction is thus able to proceed before further deprotonation.

## Prussian Blue Calculations

The lattice constant of bulk Prussian Blue (PB, $KFeCo(CN)_6$) was optmised to 9.96Å. The most favourable spin configuration was found to be 0 for both Co and Fe atoms, corresponding to Fe(II) and Co(III), in agreement with previous calculations.[17] The lattice constant of PB with ¼ $Fe(CN)_6$ defects was also optimized, revealing a similar lattice constant of 9.92Å. Potassium is removed from the unit cell for charge balance. The spin state of the metal atoms changes around the defect, such that the electronic structure is better described as Co(II), Fe(III), except from the Co atom that is not a neighbour to the defect, which remains Co(III). This is different to the results obtained in[18] however in these calculations $H_2O$ was adsorbed on the defect sites, whereas our calculations show that the adsorption of $H_2O$ is not favourable at room temperature (see below).

Prussian Blue analogues have a rich and complex defect chemistry[19] and in the following it is assumed that OER proceeds at the defect sites. However, the electronic properties of Co atoms deposited at the surface of PB crystals would probably be similar. For calculation of the OER activity a unit cell consisting of one defect-free $KFeCo(CN)_6$ unit and one unit with a $Fe(CN)_6$ vacancy was created, using the averaged lattice constant from the two bulk optimisations (see figure S15). This unit cell contains two types of exposed Co atoms; two $Co(CN)_4$ which have two neighbouring defect sites and two $Co(CN)_5$ which have only one neighbouring defect site. The adsorption of $H_2O$ on the two $Co(CN)_5$ sites is calculated to be 0.41 eV, showing that the bare catalyst is the most stable reference.

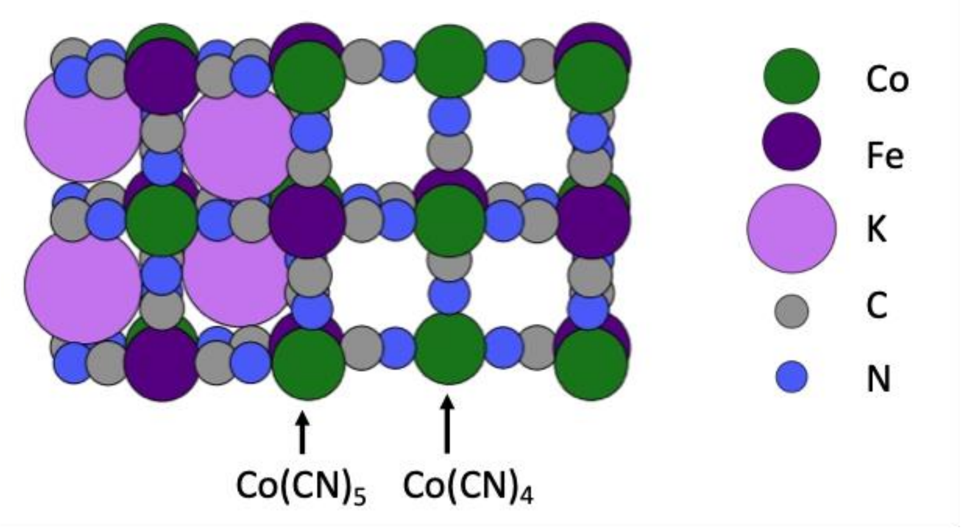


*Figure S15: Computational unit cell used to model OER in the CoFe PB analogue. The two different types of Co active sites around the defect site are marked.*

The adsorption energies of *OH and *O on the two types of Co sites are given in Table S3. Adsorption on $Co(CN)_5$ is found to be more favorable than on $Co(CN)_4$, and *OH is adsorbed on both of these sites before adsorption on the $Co(CN)_4$ sites begins. Note that the structure is calculated with no restrictions on the spin and with several different values of fixed total magnetic moment in order to find the lowest energy spin state, which is also given in Table S3.

*Table S3: Differential adsorption energies of OH on PB sites (in eV) and total spin of the structure.*

| Total no. of OH | Ads on $Co(CN)_4$ | Ads. On $Co(CN)_5$ | Total spin [$\mu_B$] | Total Eads | Eads of last OH [eV] |
|---|---|---|---|---|---|
| 1 | 1xOH | - | 7 | 1.84 | 1.84 |
| 1 | - | 1xOH | 7 | 1.40 | 1.40 |
| 2 | - | 2xOH | 6 | 2.93 | 1.54 |
| 2 | 1xOH | 1xOH | 8 | 3.26 | 1.86 |
| 3 | 1xOH | 2xOH | 5 | 4.80 | 1.87 |
| 4 | 2xOH | 2xOH | 4 | 6.45 | 1.65 |
| 5 | 3xOH | 2xOH | 5 | 8.26 | 1.81 |
| 6 | 4xOH | 2xOH | 6 | 9.91 | 1.65 |

To calculate the overpotential for OER the adsorption energy of *O is also calculated. We focus on the $Co(CN)_5$ sites, since they have the lowest *OH adsorption energies, considering *O adsorption both before and after the adsorption of the second *OH. The results shown in Table S4 show that adsorption of *O happens at a potential of 1.72-1.75 V, i.e. after the adsorption of the second *OH on $Co(CN)_5$ but before the adsorption of *OH on the $Co(CN)_4$. To ensure that OER proceeds before the adsorption on *OH on $Co(CN)_4$ the energy of *OOH on the $Co(CN)_5$ site (with and without *OH on the other $Co(CN)_5$ site) is also calculated. The resulting energy diagram in Figure S16, shows that the limiting step in the reaction is the conversion of *OH to *O and the presence of *OH on the other $Co(CN)_5$ site results in a destabilisation of all the reaction intermediates but a very similar overpotential.

*Table S4: Differential adsorption energies of O on PB sites (in eV) and total spin of the structure.*

| Total number of adsorbates | Ads. on $Co(CN)_5$ | Total spin [$\mu_B$] | Total Eads | *OH → *O [eV] |
|---|---|---|---|---|
| 1 | 1xO | 8 | 3.13 | 1.73 |
| 2 | 1xO+1xOH | 5.9/7.0[a] | 4.65 | 1.72 |
| 2 | 2xO | 7.6 | 6.41 | 1.75 |

[a] The two spin states were within 0.01 eV of each other

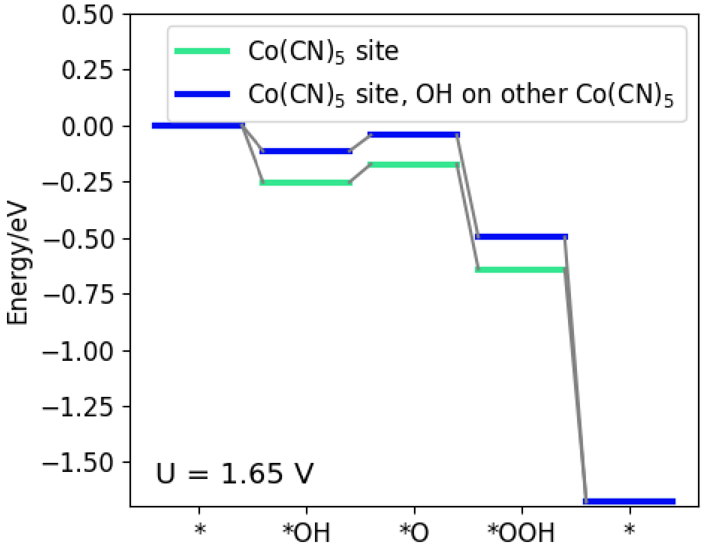


Figure S16: Energy diagram for OER at 1.65V on the $Co(CN)_5$ site of PB with and without OH adsorbed on the other $Co(CN)_5$ site.

Together these results suggest that only $Co(CN)_5$ sites are covered by adsorbates below a potential of 1.86 V, and it is thus the adsorption on these sites that give rise to the observed isotherms.

With only two adsorption sites of this type in the unit cell there are also only two different adsorption energies. However, one can imagine that the different values arise either from interactions across the vacuum at the vacancy or as a result of electronic effects through the bulk crystal. We therefore tried to calculate the adsorption energies in two other unit cells, one consisting of two bulk units and one defect (with two $Co(CN)_5$ units) and another one consisting of three bulk units and one defect in a 2x2 arrangement (with four $Co(CN)_5$ units). The calculated adsorption energies of *OH are given in Table S5, and span a range of 1.40-1.54 eV, indicating an interaction parameter of 0.14 eV. This matches well with the experimentally observed redox 2 transition. A corresponding investigation of the *O adsorption energies (Table S6) reveals an energy range of 1.68-1.76eV, matching well with the observed redox 3 ($E_0$ = 1.66V, r=0.19 eV).

| Total no. of OH | Unit cell | Ads. On $Co(CN)_5$ | Total spin [$\mu_B$] | Total Eads | Eads of last OH [eV] |
|---|---|---|---|---|---|
| 1 | 1 bulk + 1 defect | 1xOH | 7 | 1.40 | 1.40 |
| 2 | 1 bulk + 1 defect | 2xOH | 6 | 2.93 | 1.54 |
| 1 | 2 bulk + 1 defect | 1xOH | 6.5 | 1.54 | 1.54 |
| 2 | 2 bulk + 1 defect | 2xOH | 5.4 | 3.03 | 1.49 |
| 1 | 3 bulk + 1 defect | 1xOH | 9 | 1.42 | 1.42 |
| 2 | 3 bulk + 1 defect | 2xOH[a] | 8 | 2.81 | 1.40 |
| 3 | 3 bulk + 1 defect | 3xOH | 7 | 4.34 | 1.52 |
| 4 | 3 bulk + 1 defect | 4xOH | 6 | 5.80 | 1.47 |

[a] Adsorption on nearest neighbour sites is found to be the most stable arrangement.

Table S5: Differential adsorption energies of OH on $Co(CN)_5$ sites (in eV) and total spin of the structure for different sized unit cells.

| Total no. of OH | Unit cell | Ads. On $Co(CN)_5$ | Total spin [$\mu_B$] | Total Eads | Eads of last O [eV] |
|---|---|---|---|---|---|
| 1 | 1 bulk + 1 defect | 1xOH + 1xO | 5.9/7.0[a] | 4.65 | 1.72 |
| 2 | 1 bulk + 1 defect | 2xO | 7.6 | 6.41 | 1.75 |
| 1 | 2 bulk + 1 defect | 1xOH + 1xO | 3.4 | 4.71 | 1.68 |
| 2 | 2 bulk + 1 defect | 2xO | 7.5 | 6.47 | 1.76 |
| 1 | 3 bulk + 1 defect | 3xOH + 1xO | 7 | 7.55 | 1.75 |
| 2 | 3 bulk + 1 defect | 2xOH + 2xO[b] | 6.5 | 9.27 | 1.72 |
| 3 | 3 bulk + 1 defect | 1xOH + 3xO | 7.6 | 10.99 | 1.72 |
| 4 | 3 bulk + 1 defect | 4xO | 8.7 | 12.73 | 1.74 |

*Table S6: Differential adsorption energies of O on $Co(CN)_5$ sites (in eV) and total spin of the structure for different sized unit cells.*

[a] The two spin states were within 0.01 eV of each other

[b] Identical adsorbates on opposite sides of the defect is found to be the most stable arrangement


1. Burke, M. S., Kast, M. G., Trotochaud, L., Smith, A. M. & Boettcher, S. W. Cobalt–Iron (Oxy)hydroxide Oxygen Evolution Electrocatalysts: The Role of Structure and Composition on Activity, Stability, and Mechanism. *J Am Chem Soc* **137**, 3638–3648 (2015).

2. Han, L. *et al.* Enhanced Activity and Acid pH Stability of Prussian Blue-type Oxygen Evolution Electrocatalysts Processed by Chemical Etching. *J Am Chem Soc* **138**, 16037–16045 (2016).

3. Trotochaud, L., Young, S. L., Ranney, J. K. & Boettcher, S. W. Nickel-iron oxyhydroxide oxygen-evolution electrocatalysts: the role of intentional and incidental iron incorporation. *J Am Chem Soc* **136**, 6744–53 (2014).

4. Yeo, B. S. & Bell, A. T. Enhanced Activity of Gold-Supported Cobalt Oxide for the Electrochemical Evolution of Oxygen. *J Am Chem Soc* **133**, 5587–5593 (2011).

5. Moysiadou, A., Lee, S., Hsu, C.-S., Chen, H. M. & Hu, X. Mechanism of Oxygen Evolution Catalyzed by Cobalt Oxyhydroxide: Cobalt Superoxide Species as a Key Intermediate and Dioxygen Release as a Rate-Determining Step. *J Am Chem Soc* **142**, 11901–11914 (2020).

6. Enman, L. J. *et al.* Operando X-Ray Absorption Spectroscopy Shows Iron Oxidation Is Concurrent with Oxygen Evolution in Cobalt–Iron (Oxy)hydroxide Electrocatalysts. *Angewandte Chemie Int Ed* **57**, 12840–12844 (2018).

7. Kresse, G. & Hafner, J. Ab initio molecular dynamics for liquid metals. *Phys. Rev. B* **47**, 558–561 (1993).

8. Larsen, A. H. *et al.* The atomic simulation environment—a Python library for working with atoms. *J. Phys.: Condens. Matter* **29**, 273002 (2017).

9. Hammer, B., Hansen, L. B. & Nørskov, J. K. Improved adsorption energetics within density-functional theory using revised Perdew-Burke-Ernzerhof functionals. *Phys. Rev. B* **59**, 7413–7421 (1999).

10. Nørskov, J. K. *et al.* Origin of the Overpotential for Oxygen Reduction at a Fuel-Cell Cathode. *J. Phys. Chem. B* **108**, 17886–17892 (2004).

11. Moss, B. *et al.* Unraveling Charge Transfer in CoFe Prussian Blue Modified BiVO 4 Photoanodes. *Acs Energy Lett* **4**, 337–342 (2018).

12. Nong, H. N. *et al.* Key role of chemistry versus bias in electrocatalytic oxygen evolution. *Nature* **587**, 408–413 (2020).

13. Dionigi, F. *et al.* In-situ structure and catalytic mechanism of NiFe and CoFe layered double hydroxides during oxygen evolution. *Nat Commun* **11**, 2522 (2020).

14. Bajdich, M., García-Mota, M., Vojvodic, A., Nørskov, J. K. & Bell, A. T. Theoretical Investigation of the Activity of Cobalt Oxides for the Electrochemical Oxidation of Water. *J Am Chem Soc* **135**, 13521–13530 (2013).

15. Mefford, J. T. *et al.* Correlative operando microscopy of oxygen evolution electrocatalysts. *Nature* **593**, 67–73 (2021).

16. Nørskov, J. K. Fundamental ConCepts in Heterogeneous Catalysis.

17. Hegner, F. S. *et al.* Understanding the Catalytic Selectivity of Cobalt Hexacyanoferrate toward Oxygen Evolution in Seawater Electrolysis. *ACS Catal.* **11**, 13140–13148 (2021).

18. Hegner, F. S. Experimental and theoretical investigation of Prussian blue-type catalysts for artificial photosynthesis. (Universitat Rovira i Virgili http://hdl.handle.net/10803/666291, 2019).

19. Simonov, A. *et al.* Hidden diversity of vacancy networks in Prussian blue analogues. *Nature* **578**, 256–260 (2020).
